# A transposable element insertion in the susceptibility gene *CsaMLO8* results in hypocotyl resistance to powdery mildew in cucumber

**DOI:** 10.1186/s12870-015-0635-x

**Published:** 2015-10-09

**Authors:** Jeroen A. Berg, Michela Appiano, Miguel Santillán Martínez, Freddy WK Hermans, Wim H. Vriezen, Richard GF Visser, Yuling Bai, Henk J. Schouten

**Affiliations:** Wageningen UR Plant Breeding, Wageningen University & Research centre, Droevendaalsesteeg 1, 6708 PB Wageningen, The Netherlands; Bayer Crop Science Vegetable Seeds, P.O. Box 4005, 6080 AA Haelen, The Netherlands

**Keywords:** Powdery mildew, *MLO*, Susceptibility gene, Cucumber (*Cucumis sativus* L.), Hypocotyl resistance, Non-autonomous transposable element

## Abstract

**Background:**

Powdery mildew (PM) is an important disease of cucumber (*Cucumis sativus* L.). *CsaMLO8* was previously identified as a candidate susceptibility gene for PM in cucumber, for two reasons: 1) This gene clusters phylogenetically in clade V, which has previously been shown to harbour all known *MLO*-like susceptibility genes for PM identified in dicot species; 2) This gene co-localizes with a QTL on chromosome 5 for hypocotyl-specific resistance to PM.

**Methods:**

*CsaMLO8* alleles from susceptible and resistant cucumber were cloned and transformed to *mlo*-mutant tomato. Cucumber seedlings were inoculated with *Podosphaera xanthii*, tissues were studied for CsaMLO8 expression at several timepoints post inoculation using qRT-PCR. The occurence of the observed loss-of-function allele of *CsaMLO8* in resequenced cucumber accessions was studied *in silico*.

**Results:**

We cloned *CsaMLO8* alleles from susceptible and resistant cucumber genotypes, the latter carrying the QTL for hypocotyl resistance. We found that insertion of a non-autonomous Class LTR retrotransposable element in the resistant genotype leads to aberrant splicing of *CsaMLO8* mRNA. Heterologous expression of the wild-type allele of *CsaMLO8* in a tomato *mlo-*mutant restored PM susceptibility. However, heterologous expression of the *CsaMLO8* allele cloned from the resistant cucumber genotype failed to restore PM susceptibility. Furthermore we showed that inoculation of susceptible cucumber with the PM pathogen *Podosphaera xanthii* induced transcriptional upregulation of *CsaMLO8* in hypocotyl tissue, but not in cotyledon or leaf tissue. This coincides with the observation that the QTL at the *CsaMLO8-*locus causes full resistance in hypocotyl tissue*,* but only partial resistance in cotyledons and true leafs. We studied the occurrence of the loss-of-function allele of *CsaMLO8* in cucumber germplasm by an *in silico* approach using resequencing data of a collection of 115 cucumber accessions, and found that this allele was present in 31 out of 115 accessions.

**Conclusions:**

*CsaMLO8* was characterised as a functional susceptibility gene to PM, particularly in the hypocotyl where it was transcriptionally upregulated upon inoculation with the PM pathogen *P. xanthii.* A loss-of-function mutation in *CsaMLO8* due to the insertion of a transposable element was found to be the cause of hypocotyl resistance to PM. This particular allele of *CsaMLO8* was found to occur in 27 % of the resequenced cucumber accessions.

**Electronic supplementary material:**

The online version of this article (doi:10.1186/s12870-015-0635-x) contains supplementary material, which is available to authorized users.

## Background

Cucumber (*Cucumis sativus* L.) is an economically important crop, with an annual global production of over 65 megatons [1]. Powdery mildew (PM) is one of the most widespread diseases in cucurbits, and a limiting factor for cucumber production. Two species of fungi have been reported to cause PM in cucumber, i.e., *Podosphaera xanthii* (synonymous with *P. fusca*, previously named *Sphaerotheca fuliginea*) and *Golovinomyces cichoracearum* (previously named *Erysiphe cichoracearum*). Of these, *P. xanthii* is considered to be the main causal agent of PM in cucurbits [2, 3].

Breeding of resistant cucumber varieties has been undertaken for several decennia (e.g., [4–6]), but underlying resistance genes have to date not been functionally characterised. As the genome of cucumber (‘Chinese long’ inbred line 9930) was published in 2009 [7], and several other cucumber accessions have been resequenced [8, 9], the time is now ripe to identify causal genes for cucumber resistance to mildew diseases.

Traditionally, breeding of disease resistant crops is performed by introgression of resistance (*R*) genes, often from wild relatives of the crop. R proteins, most commonly of the nucleotide-binding, leucine-rich-repeat (NB-LRR) type, are able to recognise either corresponding avirulence (*Avr*) gene products of the pathogen, or degradation products of host factors associated with pathogen attack [10]. This triggers a defence response in the host cell, often associated with a hypersensitive response (HR), leading to cell death [10]. As *R* genes recognise very specific products, introgression and subsequent employment of a new *R* gene puts selective pressure on the pathogen to evolve in such a way that it is no longer recognised by the host plant. Therefore, *R*-gene based resistance is often breached by new, virulent, races of the pathogen quite soon, especially for versatile pathogens, such as powdery mildew fungi [10].

An alternative for *R*-gene mediated resistance is the identification of impaired susceptibility (*S*) genes [11]. Most pathogens require cooperation of their host plant to be able to successfully establish a compatible interaction [12]. This is especially true for biotrophic pathogens such as mildew species, as they greatly rely on a long-lasting interaction with (living) host cells to facilitate their propagation [12]. Therefore, the expression of several host genes is essential for the pathogen. Such genes can be regarded as *S* genes, and can function for instance in facilitating host recognition and penetration, negative regulation of host defences or fulfilling metabolic and structural needs of the pathogen [12]. Loss-of-function mutations in a *S* gene are thought to lead to durable, broad spectrum, recessively inherited resistance [13, 14].

The barley *mlo* gene is one of the best-known examples of an impaired *S* gene. After it first was found in the 1940s in a mutagenized barley population [15], deployment of loss-of-function *mlo* alleles in barley has resulted in PM resistant barley varieties. These have been grown in the field for several decades already without breaching of resistance by virulent new mildew races to date, providing evidence for the durability of *S*-gene based resistance [16]. After the barley *MLO* gene was cloned [17], it was found that *MLO* genes are conserved throughout the plant kingdom and occur in higher plants as a multi-copy gene family [18, 19]. In several plant species, *MLO*-like genes have been found to be involved in PM susceptibility, such as *Arabidopsis*, tomato, pea, pepper, tobacco, bread wheat and potentially also grapevine and peach [20–27]. It has been found that in phylogenetic trees of the *MLO* gene family all *MLO-*like S-genes for PM detected in monocotyledonous species cluster in clade IV, whereas all *MLO-*like *S*-genes identified in dicotyledonous species cluster in clade V. The other clades (I, II, III and VI) harbour *MLO-*like genes that have not been proven to be *S*-genes [19].

The genome of cucumber harbours 13 putative *MLO-*like genes [28]. Of these, three (i.e., *CsaMLO1*, *CsaMLO8* and *CsaMLO11*,with respective Cucurbit Genomics Database IDs [Csa1M085890.1], [Csa5M623470.1] and [Csa6M292430.1]) cluster in clade V of the *MLO* gene family, and can therefore be considered candidate *S*-genes for powdery mildew resistance [28]. *CsaMLO8* is of particular interest, as its position on the genome (Chr5: 24,827,408..24,831,456) co-localizes with *pm5.2*, a recently identified major QTL explaining 74.5 % of the phenotypic variation for ‘hypocotyl’ resistance in F3 families derived from the resistant cucumber inbred line WI 2757 [29]. ‘Hypocotyl’ or intermediate resistance of cucumber to PM was previously shown to be a recessively inherited monogenic trait in crossings between several cucumber lines, and was characterised by completely resistant hypocotyl, stem and petiole tissue and partially resistant leaves and cotyledons. Hypocotyl resistance is suggested to play an important role in overall PM resistance of cucumber, as it appears that complete resistance in leaves is not possible without the recessive hypocotyl resistance gene [5]. In breeding practice loss of the hypocotyl resistance allele leads to PM susceptible seedlings. The allele is present in almost all modern pickling cucumber varieties, and most of the resistant long cucumber varieties (Freddy Hermans, personal communications), showing the agricultural significance of hypocotyl resistance in cucumber.

Here, we report the cloning of *CsaMLO8* from both susceptible and (hypocotyl) resistant cucumber genotypes. We show that at the transcript level the allele obtained from the resistant genotype has deletions of 72 or 174 bp due to alternative splicing, caused by the insertion of a LTR retrotransposable element in this gene at the genomic level. Complementation of the tomato *mlo*-mutant with the wild-type and ∆174 alleles of *CsaMLO8* showed that wild-type *CsaMLO8* is a functional susceptibility gene (*S*-gene), whereas the ∆174 allele has lost its function as *S*-gene, thus leading to PM resistance. Furthermore, qRT-PCR showed that *CsaMLO8* is transcriptionally upregulated upon inoculation with *P. xanthii* in hypocotyl tissue, but not in leaves or cotyledon, explaining why loss-of-function of *CsaMLO8* provides particularly resistance in the hypocotyl.

## Results

### Cloning and sequencing of the *CsaMLO8* coding sequence from susceptible and resistant genotypes

**Fig. 1:**
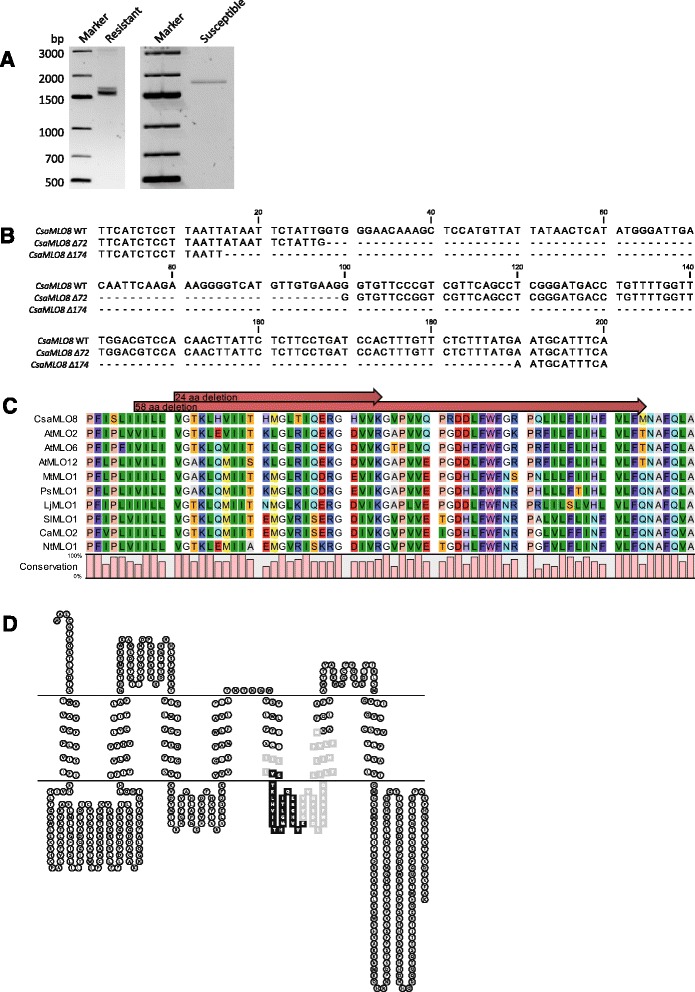
Characterization of *CsaMLO8* alleles from resistant and susceptible cucumber genotypes. **a** cDNA of resistant (left panel) and susceptible (right panel) cucumber genotypes was used as template for PCR with *CsaMLO8* specific primers. Amplified products were analysed on 1.25 % agarose gels. Whereas the product amplified from cDNA of the susceptible genotype gives a single band of the expected size, cDNA of the resistant genotype results in two separate bands, both of a smaller size than expected. **b** Full length *CsaMLO8* amplified from cDNA from susceptible and resistant cucumber genotypes was sequenced. A partial alignment is shown between the (wild-type) sequence as obtained from the susceptible genotype and the sequences from two deletion variants (∆72 and ∆174) obtained from the resistant genotype. Numbers are relative to the start of the alignment. **c** Partial alignment of the CsaMLO8 protein and other proteins encoded by clade V *MLO S-*genes of several species. Amino acid residues are coloured according to the RasMol colour scheme. The 24 and 58 amino acid residues deleted in the proteins encoded by the ∆72 and the ∆174 variants of *CsaMLO8* are indicated by red arrows. A bar graph underneath the alignment indicates the conservedness of each amino acid position. **d** Graphic representation of the transmembrane structure of the predicted CsaMLO8 protein, determined using HMMTOP 2.1 [30]. The plasma membrane is indicated by two horizontal lines. Amino acid residues highlighted in black are predicted to be deleted in the protein encoded by the ∆72 variant of the *CsaMLO8* gene, residues highlighted in black and grey are predicted to be deleted in the protein encoded by the ∆174 variant of the *CsaMLO8* gene

We performed RT-PCR using RNA derived from either a susceptible wild-type cucumber cultivar or a resistant breeding line known to be homozygous for the *hypocotyl resistance* QTL as a template. Whereas the product we obtained from the susceptible genotype was of the expected size (i.e., 1726 bp), we obtained two different products from the resistant genotype, both smaller than expected (Fig. 1a). Sequence analysis revealed that the *CsaMLO8* mRNA variant obtained from the susceptible genotype was identical to the predicted coding sequence. The two mRNA products obtained from the resistant genotype however had (non-frameshift) deletions of respectively 72 and 174 bp. The 174 bp deletion variant corresponds to a loss of the complete 11th exon of the *CsaMLO8* gene, whereas the 72 bp deletion variant corresponds to the loss of a fragment of the 11th exon with canonical splice sites (5′-GT and AG-3′) (Fig. 1b). Furthermore, the coding sequence of the resistant genotype has five (synonymous) SNPs compared to the reference genome (Additional file 1).

To determine the impact of the 72 and 174 bp deletions found in the mRNA on the predicted CsaMLO8 protein sequence, the predicted CsaMLO8 protein was aligned to a dataset of MLO proteins encoded by clade V S-genes from several other species i.e., *Arabidopsis*, barrel clover, pea, lotus, tomato, pepper and tobacco (Additional file 2). It appeared that the region encoded by the deleted area in the 72 and 174 bp deletion variants is highly conserved among different MLO proteins (Fig. 1c). Furthermore, the transmembrane structure of the CsaMLO8 protein (wild-type allele) was predicted using HMMTOP 2.1 software [30]. The predicted transmembrane structure of the wild-type protein was largely consistent with the barley MLO structure determined by Devoto et al. [18, 19]. The 72 and 174 bp deletions correspond to removal of a region of 24 respectively 58 amino acid residues in the (predicted) third cytoplasmic loop of CsaMLO8 (Fig. 1d).

The relative transcript abundances of the two *CsaMLO8* splice variants characterised by the 72 and 174 bp deletions were determined by qRT-PCR using splice junction spanning primers on different tissues (i.e., hypocotyl, cotyledon and true leaf) of PM resistant cucumber, either inoculated with PM or non-inoculated. It appeared that the 174 bp deletion splice variant was the most abundant isoform, whereas the 72 bp deletion splice variant was less abundant in each tissue regardless whether tissues were inoculated or not (Additional file 3).

### Complementation of *SlMLO1* loss-of-function tomato mutant with *CsaMLO8* WT and *CsaMLO8∆174*

The sequence analysis of the transcripts of *CsaMLO8* from susceptible and resistant genotypes led to the hypothesis that *CsaMLO8* is a functional *S-*gene for PM, whereas the 174 bp deletion allele (*CsaMLO8∆174*) has lost its function as *S-*gene. To test these hypotheses, both alleles were overexpressed in a previously described tomato *mlo*-mutant, which carries a mutation in the tomato *SlMLO1* gene and is resistant to tomato powdery mildew, *Oidium neolycopersici* [21].

**Fig. 2:**
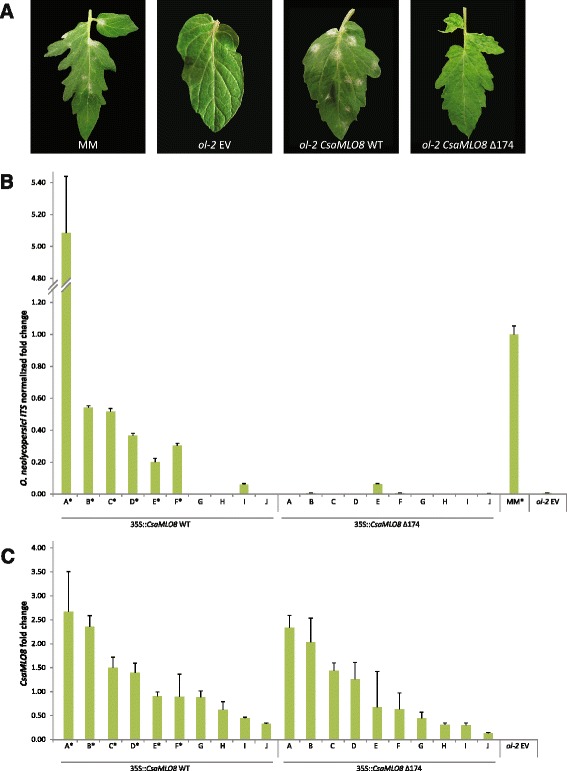
Complementation of *ol-2* tomato with *CsaMLO8* WT restores PM susceptibility, whereas complementation with *CsaMLO8∆174* does not. The PM resistant *ol-2* tomato mutant with a deletion in *SlMLO1* [21] was transformed with either a 35S::*CsaMLO8* WT construct, a 35S::*CsaMLO8∆174* construct, or an empty vector (EV) control. Cuttings from these transformants were inoculated with a *Oidium neolycopersici* spore suspension. As additional control we used the wild-type, susceptible cv. Moneymaker (MM). **a** The phenotype of susceptible control MM, resistant EV transformed *ol-2,* and transgenic individuals overexpressing either *CsaMLO8* WT or *CsaMLO8∆174* in *ol-2* background. Photographs were taken 16 days post inoculation. **b** Relative quantification by qPCR of the ratio between *Oidium neolycopersici* and plant gDNA in susceptible MM, resistant EV transformed *ol-2,* and transgenic individuals overexpressing either *CsaMLO8* WT or *CsaMLO8∆174* in *ol-2* background. Fold changes were normalised relative to the susceptible control MM. Bars represent the average fold change over 3 technical replicates. Error bars indicate standard deviation. Asterisks indicate plants scored as susceptible to powdery mildew based on macroscopic evaluation. **c** Relative quantification by qRT-PCR of the ratio between *CsaMLO8* expression and expression of tomato housekeeping gene *SlEF-α* in EV transformed *ol-2* and transgenic individuals overexpressing either *CsaMLO8* WT or *CsaMLO8∆174* in *ol-2* background. Bars represent the average fold change over 3 technical replicates. Error bars indicate standard deviation. Asterisks indicate plants scored as susceptible to powdery mildew based on macroscopic evaluation

Cuttings of ten independent transgenic individuals per construct (*35S::CsaMLO8* WT and *35S::CsaMLO8∆174*) were challenged with the tomato PM pathogen *O. neolycopersici*. Powdery mildew susceptibility was evaluated qualitatively, by looking for PM symptoms on the leaves (Fig. 2a, Additional file 4). Six out of ten individual transformants expressing *CsaMLO8* WT were scored as susceptible to PM, whereas none of the transformants expressing *CsaMLO8∆174* were scored as susceptible to PM. PM susceptibility was confirmed quantitatively, by performing qPCR on DNA isolated from inoculated leaves, using *O. neolycopersici* specific primers. This showed that the biomass of *O. neolycopersici* in plants scored as susceptible to PM was at least 0.20, relative to the biomass in the susceptible control MM, whereas the biomass in plants scored as resistant was less than 0.20 (Fig. 2b). Furthermore, transcript abundances of the transgenes in each of the transgenic individuals were determined by qRT-PCR using *CsaMLO8* specific primers (Fig. 2c). This confirmed that transcript levels of *CsaMLO8* WT and *CsaMLO8∆174* were comparable. The six *CsaMLO8* WT transformants scored as susceptible to PM had a higher *CsaMLO8* expression than the four *CsaMLO8* WT transformants scored as resistant to PM.

### Sequencing and characterization of a transposable element in *CsaMLO8*

**Fig. 3:**
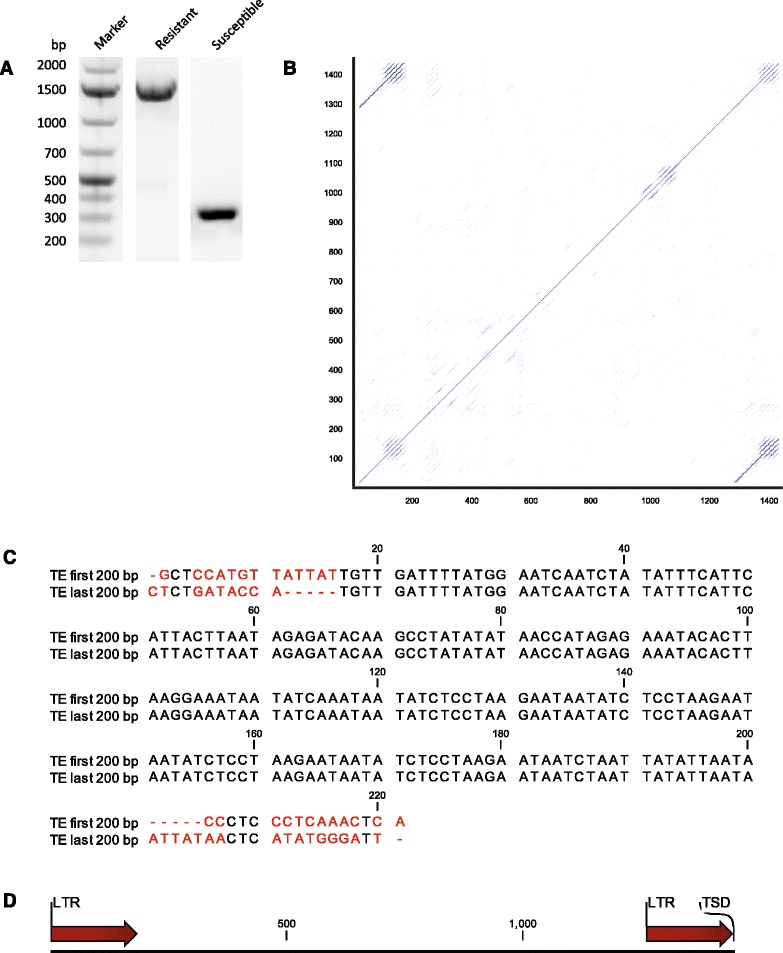
Amplification and sequencing of *CsaMLO8* from genomic DNA isolated from the resistant genotype reveals the insertion of an 1449 bp long Transposable Element (TE). **a** The genomic region of *CsaMLO8* in which deletions in the coding sequence were observed in the resistant genotype was amplified from DNA isolated from both the susceptible and resistant genotypes. Amplified products were analysed on 1.25 % agarose gel. Whereas the product amplified from the susceptible genotype was of the expected size, the product amplified from the resistant genotype was larger than expected. **b** The product amplified from the resistant genotype as described in (A) was sequenced, which revealed an insertion with a length of 1449 bp. A dot-plot was made of the insertion to see whether the sequence contains repetitive elements. **c** The first and last 200 bp of the insertion, plus 15 bp of *CsaMLO8* before and after the insertion were aligned to one another, to verify the presence of long terminal repeats (LTRs). Non-aligned parts of the sequence are highlighted in red. It can be seen that the first 184 bp of the insertion are completely identical to the last 184 bp of the insertion. There is a duplication of 5 bp from *CsaMLO8* before and after the insertion (Target site duplication, 5′-ATTAT-3′). **d** Schematic representation of the insertion. The locations of LTRs and the 3′ TSD are indicated

To investigate the cause of the deletions in the *CsaMLO8* coding sequence, we performed PCR using DNA from both the susceptible and resistant cucumber genotypes as a template, with primers designed to amplify the region that contained the deletions in *CsaMLO8*. The product amplified from the susceptible genotype had the expected size (i.e. 346 bp), whereas the product amplified from the resistant genotype was larger (ca. 1500 bp, Fig. 3a). Sequence analysis of the amplified product revealed a 1449 bp insertion in the genomic DNA sequence of the resistant genotype compared to the susceptible genotype. This insertion in the DNA of the resistant genotype coincided with the region that contained the deletion in the *CsaMLO8* mRNA of this genotype. Characterization of this genomic insertion by a dot-plot (Fig. 3b) revealed the presence of long terminal repeats (LTRs) with a length of ca. 200 bp. An alignment between the first and last 200 bp of the insertion confirmed the presence of 184 bp long LTRs beginning with a 5′-TG-3′ and ending with a 5′-TA-3′ (Fig. 3c). The LTRs share 100 % sequence identity with one another. After the 3′ LTR, there is a duplication of the 5 bp of *CsaMLO8* before the insertion (Target Site Duplication, TSD, 5′-ATTAT-3′). No open reading frames (ORFs) could be detected in the insertion. Taken together, these findings led us to the conclusion that the insert is most likely a non-autonomous transposable element (TE) of Class I, Order LTR, according to the transposable element classification scheme proposed by Wicker et al. [31].

### Similar TEs in the cucumber genome

**Fig. 4:**
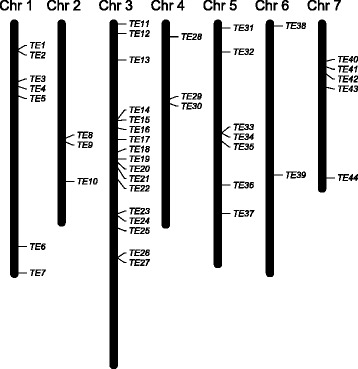
There are 44 putative homologous TEs in the cucumber reference genome. A BLASTn search was performed on the cucumber reference genomes with the LTR sequence of the TE found to be inserted in *CsaMLO8*. Pairs of putative LTRs within 20 kb of one another were considered borders of putative TEs. 44 putative TEs were identified, chromosomal locations of which are indicated

In an attempt to identify homologous, potentially autonomous, transposable elements in the cucumber genome, we performed a BLASTn search on the cucumber reference genome (Chinese long inbred line ‘9930’, v2) with the LTR sequence of the TE found in *CsaMLO8* as query. We identified 169 putative homologous LTRs. A previously designed tool [32] was used to screen the genome for regions bordered by two putative homologous LTR sequences. Two putative homologous LTR sequences within a window of 20 kb were considered to be the borders of a putative homologous TE. The 20 kb window was decided upon based on the observation that LTR retrotransposons are generally between 3 and 15 kb of size [33], the only exception to our knowledge being the very large *Ogre* retrotransposons found in legumes [34], which have ca. 5 kb LTRs and are therefore ca. 22 kb in size. A total of 44 putative TEs was identified, randomly distributed over all seven chromosomes of the cucumber reference genome (Fig. 4, Additional file 5). For 20 putative TEs, the complete sequence in between the LTRs was extracted from the genome, and compared to the sequence of the TE found in *CsaMLO8* (Additional file 6). It was found that most of the putative TEs have a length comparable to the *CsaMLO8*-TE, being between 1 and 2 kb. One putative TE was considerably larger than average, with 7142 bp, whereas one putative TE was considerably smaller than average, i.e., 367 bp. In only one out of the 20 putative TEs (TE37), an open reading frame (ORF) could be detected. This ORF, with a length of 411 bp, does not lead to a predicted protein with any similarity to known proteins according to a BLASTp search against all non-redundant protein databases, and is therefore considered a false positive ORF. We conclude that we could not detect an autonomous TE that contained the genes that could have been responsible for the insertion of the non-autonomous TE in *CsaMLO8.*

### Occurrence of the TE-allele of *CsaMLO8* in cucumber germplasm

We were interested to see how frequently the TE-allele of *CsaMLO8* we have characterised in our resistant cucumber genotype occurs in the cucumber germplasm. As Qi et al. (2013) resequenced a core collection of 115 very divergent cucumber accessions [8], we decided to perform an *in silico* search for the presence of the mutant *CsaMLO8* allele containing the transposable element TE) and/or the wild type (WT) allele among those genotypes. For 21 resequenced accessions (18 %) we could only detect reads indicating presence of the TE-allele. For 82 resequenced accessions (71 %) we could only find reads indicating presence of the WT-allele. For 10 accessions (9 %) we found reads indicating presence of both alleles. For the remaining two accessions (2 %), presence of neither of the alleles could be identified (Table 1, Additional file 7). The TE-allele of *CsaMLO8* was present in three out of the four geographic groups of accessions (i.e., East Asian, Eurasian and Indian but not Xishuangbanna) as defined by Qi et al. [8]. One of the 31 accessions in which the TE-allele of *CsaMLO8* was detected (i.e., PI 215589) belongs to the wild form of cucumber, *Cucumis sativus* var. *hardwickii,* whereas the other 30 accessions belong to the cultivated form of cucumber, *C. sativus* var. *sativus*.

**Table 1:** Thirty-one out of 115 resequenced cucumber accessions have the TE-allele of *CsaMLO8*

Accession number NCBI SRA	TE-allele reads	WT-allele reads	Putative genotype	PI or CGN number	Name accession	Group
SRR543205	9	0	Homozygous	PI 215589	13598	Indian
SRR543216	17	0	Homozygous	V05A0674	Bei Jing Xiao Ci	East Asian
SRR543221	1	9	Heterozygous	V05A1333	Liao Tong Mi Ci	East Asian
SRR543223	19	0	Homozygous	V05A0920	He Cha Huang Gua	East Asian
SRR543224	19	0	Homozygous	V05A1115	Qian Qi Li Huang Gua	East Asian
SRR543225	1	7	Heterozygous	V05A0985	Ye San Bai	East Asian
SRR543226	23	0	Homozygous	V05A0428	Liao Yang Ye San	East Asian
SRR543228	1	0	Homozygous	-	228	East Asian
SRR543230	18	0	Homozygous	V05A0522	Huang Gua	East Asian
SRR543231	5	8	Heterozygous	V05A0552	Qing Dao Qiu Ye Er San	East Asian
SRR543240	1	13	Heterozygous	CGN19828	-	East Asian
SRR543242	22	0	Homozygous	V05A0034	Da Ci Huang Gua	East Asian
SRR543243	12	1	Heterozygous	V05A1427	Qiu Huang Gua	East Asian
SRR543244	1	7	Heterozygous	V05A0291	Leng Lu Huang Gua	East Asian
SRR543246	1	0	Homozygous	-	Bai Ye San	East Asian
SRR543251	4	0	Homozygous	-	2004348	East Asian
SRR543252	11	0	Homozygous	CGN20266	Hok	Eurasian
SRR543253	6	0	Homozygous	-	151G	Eurasian
SRR543257	5	0	Homozygous	CGN20512	752	Eurasian
SRR543258	9	0	Homozygous	CGN20515	Gy 3 (S4)	Eurasian
SRR543264	2	0	Homozygous	-	65G	Eurasian
SRR543265	11	0	Homozygous	-	G8	Eurasian
SRR543267	14	0	Homozygous	V05A0726	Jin Yan Er Hao	East Asian
SRR543269	10	3	Heterozygous	CGN19579	1972 B-2	Eurasian
SRR543271	15	0	Homozygous	CGN19844	2163	Eurasian
SRR543272	2	5	Heterozygous	PI 234517/CGN20898	SC 50	Eurasian
SRR543274	11	0	Homozygous	CGN21627	Spartan Garden MSU-C7-63	Eurasian
SRR543275	4	0	Homozygous	-	Marketmore76	Eurasian
SRR543276	4	0	Homozygous	-	GY14	Eurasian
SRR543281	9	3	Heterozygous	PI 482412	TGR 580	Indian
SRR543293	6	8	Heterozygous	PI 605943	USM 307	Indian

Total reads of 115 recently resequenced cucumber accessions [8] were assayed *in silico* for the presence of reads indicating the presence of either the allele of *CsaMLO8* characterised by the insertion of a TE, or the wild-type allele. The amount of reads indicating presence of either the TE-allele or the WT-allele of *CsaMLO8* is given. Database number, accession names and geographic groups of accessions were obtained from [8]

### Inoculation with *P. xanthii* induced transcription of *CsaMLO8* in hypocotyl tissue, but not in leaf tissue of susceptible cucumber

**Fig. 5:**
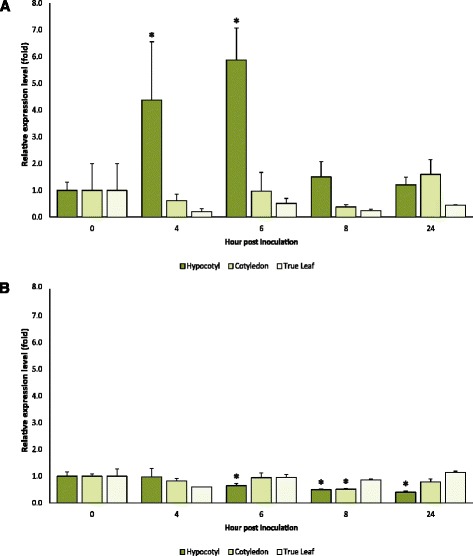
*CsaMLO8* transcription is induced after inoculation with *Podosphaera xanthii* in hypocotyl tissue, but not in cotyledon or true leaf tissue. Susceptible (**a**) and resistant (**b**) cucumber seedlings were inoculated with a *P. xanthii* spore suspension. Prior to and 4, 6, 8 and 24 h post inoculation, hypocotyl, cotyledon and true leaf tissue were harvested and immediately frozen in liquid nitrogen. Relative quantification of *CsaMLO8* expression was performed by qRT-PCR. Fold changes were normalised relative to *CsaMLO8* expression prior to inoculation. Bars represent the average fold change over three independent biological replicates. Error bars indicate standard errors of the mean. Asterisks indicate significant differences to the expression prior to inoculation (Student’s *T* test, *P* < 0.05)

*MLO* genes involved in PM susceptibility are upregulated in several plant species several hours after inoculation (e.g., [26, 35, 36]). To see whether the same holds true for *CsaMLO8,* we performed qRT-PCR experiments to quantify *CsaMLO8* transcript abundances in hypocotyl, cotyledon and leaf tissues of PM susceptible and resistant cucumber plants, prior to and at 4, 6, 8 and 24 h after PM inoculation (Fig. 5). For PM susceptible plants, we found that in hypocotyl tissue *CsaMLO8* transcript abundance was significantly higher at 4 hpi (*P* = 0.037) and 6 hpi (*P* = 0.004) compared to the transcript abundance prior to inoculation (0 hpi). The significant difference had disappeared 8 hpi (*P* = 0.212) and 24 hpi (*P* = 0.281). Contrastingly, *CsaMLO8* transcript abundances in cotyledons and true leaves were not significantly altered at any of the evaluated time points after PM inoculation (*P* > 0.05) (Fig. 5a). For PM resistant plants, we found that *CsaMLO8* transcript abundance was not significantly higher in any tissue at any time point after inoculation compared to the transcript abundance prior to inoculation (*P* > 0.05). In hypocotyl tissue, transcript abundance was significantly lower at 6 hpi (*P* = 0.046), 8 hpi (*P* = 0.006) and 24 hpi (*P* = 0.009) compared to the transcript abundance prior to inoculation (0 hpi). In cotyledon tissue, transcript abundance was significantly lower at 8 hpi (*P* = 0.002) compared to the transcript abundance prior to inoculation (Fig. 5b).

## Discussion

### *CsaMLO8* is a functional susceptibility gene for PM in cucumber

Several studies characterised some, but not all, clade V *MLO* genes as being required for PM susceptibility in different dicotyledonous plant species [20–23, 25–27]. Here we have shown that heterologous expression of the cucumber gene *CsaMLO8* in *Slmlo1* mutant tomato background restored PM susceptibility, providing evidence for the role of *CsaMLO8* as a susceptibility gene for PM in cucumber (Fig. 2). As the role of clade V *MLO* genes in susceptibility to PM seems to be evolutionary conserved between divergent dicotyledonous plant families, e.g., Brassicaceae [20], Solanaceae [21, 23, 25], Fabaceae [22], Vitaceae [26], Rosaceae [27, 36] and now also Cucurbitaceae, it is probable that in other economically important species belonging to the family Cucurbitaceae, such as melon (*Cucumis melo*) and pumpkin (*Cucurbita pepo*) clade V *MLO* genes will also play a role in PM susceptibility. Indeed, in a patent application a functional complementation of *Arabidopsis Atmlo2*, *Atmlo2,6* and *Atmlo2,6,12* mutants by a melon *MLO*-like gene was claimed to partially restore PM susceptibility, based on the percentage of diseased leaf area in 4 to 9 primary transformants [37]. Alignment of this melon *MLO* gene with the three Clade V genes of cucumber revealed that the gene from melon is most similar to *CsaMLO8,* and less alike to the two other Clade V genes (i.e., *CsaMLO1* and *CsaMLO11*) [28]. This is consistent with our finding that *CsaMLO8* is a *S-*gene for PM. In tomato we observed that complementation of *SlMLO1* loss-of-function mutants with *CsaMLO8* restored PM susceptibility, with individual transformants with higher *CsaMLO8* expression generally being more susceptible to PM than transformants with lower *CsaMLO8* expression (Fig. 2). It seems possible that in the case of complementation of *Arabidopsis* mutants by the melon *MLO* gene there was also a quantitative effect due to different levels of melon *MLO* expression in individual transformants, leading to the conclusion that the melon *MLO* gene only partially restores susceptibility whereas it was possibly due to the fact that transgene expression was not high enough to fully complement the loss of *AtMLO* function.

### Transposon insertion in *CsaMLO8* leads to aberrant splicing and therefore to loss of the *S*-gene function

By cloning *CsaMLO8* from cDNA of a PM resistant cucumber genotype that is homozygous for the *hypocotyl resistance* QTL, we found evidence for aberrant splicing of *CsaMLO8* in this genotype, leading to products with deletions of respectively 72 and 174 bp in exon 11, compared to the WT gene. We showed that these deletions are predicted to lead to loss of 24 respectively 58 amino acid residues in the third cytoplasmic loop of the CsaMLO8 protein, in a highly conserved region between clade V MLO proteins from different species (Fig. 1). As it was previously shown that cytoplasmic loop-loop interplay is required for MLO function [38], we anticipated that such rather big deletions in one of the cytoplasmic loops, if the protein should properly fold at all, would lead to loss-of-function of the protein. Indeed, we showed here that expression of the ∆174 variant of *CsaMLO8* in *Slmlo* mutant tomato background failed to restore PM susceptibility (Fig. 2). This makes cucumber, after barley [17], tomato [21] and pea [22], the fourth plant species in which a natural mutation in an *MLO* gene has been found to lead to resistance. Although we did not try to complement *Slmlo* mutant tomato with the 72 bp deletion variant of *CsaMLO8*, and thus cannot rule out the possibility that it is (partially) functional as an S gene, we expect that the result will be similar to the 174 bp deletion variant, given the conservedness of the deleted region.

To determine the reason for the aberrant splicing of *CsaMLO8* in the resistant cucumber genotype, we set out to amplify and sequence the genomic region of *CsaMLO8* in which the deletions were detected. In this way, we discovered a 1449 bp insertion in exon 11 of the gene compared to the reference genome. Sequence analysis of the insertion revealed the presence of 100 % identical LTRs and TSDs, but no open reading frames or any similarity to known proteins or genes (Fig. 3), leading to the conclusion that the insertion is probably a Class I, Order LTR (retro) transposable element (TE), following the TE classification scheme proposed by Wicker et al. [31]. The fact that the LTRs are completely identical to one another is an indication that the TE is relatively recently inserted. The integration of a transposable element in a *MLO* gene, leading to aberrant splicing of transcripts and in that way to loss of gene function, is reminiscent of the findings in the pea *PsMLO1* gene, where in one of the alleles (found in PM resistant pea cultivar JI 2302) the integration of an *Ogre* LTR retrotransposon lead to aberrant splicing [22].

We analysed putative TEs with similar LTRs (Fig. 4), and found no functional ORFs in these TEs, confirming that we are dealing with a family of non-autonomous TEs. Additionally, a large amount of LTR singlets (i.e., LTR sequences without a partner) were detected, as only 88 out of the 169 detected LTRs could be assigned to a putative TE (Additional file 5). LTR singlets presumably originate from the unequal recombination between two LTRs of a single element [39], or from assembling errors of the reference genome. It is known that plant genomes are to a great extent shaped by the integration of large amounts of transposable elements, with LTR retrotransposons being the most abundant among them (e.g., [40, 41]). The cucumber genome was shown to be no exception to this, with 24 % of the genome consisting of transposable elements and LTR retrotransposons comprising 10.4 % of the genome [7]. To our knowledge, the TE we found to be inserted in *CsaMLO8* is the first TE with a reported effect on a cucumber gene. It seems likely that more TEs with an effect on genes in cucumber will be found in the future.

### *CsaMLO8* is upregulated upon *P. xanthii* inoculation in hypocotyl tissue only

Resistance to PM in cucumber has previously been reported to be tissue specific, with an important, recessively inherited gene providing full PM resistance in hypocotyl tissue and partial resistance in leafs [5]. Recently, PM resistance of cucumber was mapped in multiple tissues separately. The strongest QTL for hypocotyl resistance, *pm5.2* was mapped on chromosome 5, in a region containing *CsaMLO8* [29]. In this study, we showed that *CsaMLO8* was, in susceptible cucumber, transcriptionally upregulated in hypocotyl tissue at 4 and 6 h post inoculation, but not in cotyledon or leaf samples (Fig. 5a). Apparently, the ability of the pathogen to upregulate *CsaMLO8* expression is specific for hypocotyl tissue. Therefore, we postulate that it is very well possible that PM resistance caused by a loss of function allele of *CsaMLO8* would also be specific for hypocotyl tissue.

Interestingly, *CsaMLO8* was not found to be transcriptionally upregulated in hypocotyl tissue (or any other tissue) in the resistant cucumber line (Fig. 5b). This is in sharp contrast with the findings in barley [35] where transcription of the *MLO* gene seemed to be even stronger induced upon PM inoculation in *mlo* loss-of-function mutants compared to wild type plants. In tomato it was found that transcription of the *SlMLO1* gene was slightly upregulated upon PM inoculation in *slmlo1* loss of function mutants, but to a far lesser extent than in wild type plants [21]. Although it remains a question why the pathogen is unable to upregulate *CsaMLO8* expression in our resistant cucumber line several explanations might be offered, e.g., lesser transcript stability of the mutant *CsaMLO8* transcripts, differences in the promotor region of the mutant allele of *CsaMLO8* or differences in other genes required for *CsaMLO8* expression compared to the susceptible cultivar.

Previously, RNA-seq experiments on cucumber leaf tissue revealed that of the thirteen *CsaMLO* genes only *CsaMLO1*, another clade V *MLO* gene, was transcriptionally upregulated after inoculation with *P. xanthii* [28]. This is consistent with our finding that *CsaMLO8* is not upregulated in leaf samples after PM inoculation (Fig. 5). It is possible that *CsaMLO1* and *CsaMLO8* are functionally redundant, but are specifically expressed in separate tissues (i.e., *CsaMLO1* specific in leaf tissue and *CsaMLO8* in hypocotyl tissue). To our knowledge there are no other examples of tissue specialization in *MLO-*like *S* genes of other species. In *Arabidopsis*, which also has three clade V *MLO* genes, *Atmlo2* mutants were found to be partially resistant, double mutants *Atmlo2*/*Atmlo6* or *Atmlo2*/*Atmlo12* were more resistant than *Atmlo2* single mutants, and triple mutants *Atmlo2*/*Atmlo6*/*Atmlo12* were completely resistant [20]. It is not yet known by what mechanism *MLO* genes are transcriptionally upregulated upon PM infection, although it would seem intuitive to hypothesise that it is an active process caused by an effector of the fungus. Given the tissue specificity of *MLO* upregulation in cucumber, this might be an interesting model to investigate the mechanism of *MLO* upregulation by PM fungi.

### The transposon insertion allele of *CsaMLO8* occurs frequently in cucumber germplasm

Interestingly, during the preparation of this manuscript, another group reported the fine-mapping of a QTL for PM resistance on the long arm of chromosome 5, which they called *pm5.1*, to a region of 170 kb containing 25 predicted genes. The main candidate gene in this region was found to be a *MLO* like gene, which appears to be the same as *CsaMLO8* in our study. By cloning and sequencing of this gene from genomic DNA of their resistant parent, line S1003, as well as two additional unrelated resistant lines, S02 and S06, they found that they contained a 1449 bp insert in the 11th exon of the gene [42]. Sequence analysis indicates that the location and sequence of the insertion found in their study are completely identical to the LTR retrotransposon described in this study. These researchers did not report on cloning the coding sequence of *CsaMLO8* in their material, nor on complementation experiments.

Additionally, a patent was filed describing an allele of *CsKIP2*, a gene claimed to provide PM resistance, shown to harbour a 72 bp deletion in the coding sequence [43]. Although it is not shown in the patent, the occurrence of this allele is claimed to be caused by the integration of a transposon-like element in the 11th exon of the gene. Sequence analysis revealed that *CsKIP2* is in fact the same gene as *CsaMLO8*, and the 72 bp deletion allele they describe is the same as the 72 bp deletion we found in our material. Interestingly the patent does not describe the 174 bp deletion which we found, but an *in silico* prediction showed that the 174 bp deletion variant would not be amplified by the primers they chose to amplify the partial *CsaMLO8* sequence. In the patent no functional proof is given that this allele of *CsaMLO8* indeed leads to resistance.

As several groups independently found the same allele of *CsaMLO8* in different, to our knowledge unrelated, resistant cucumber genotypes, we were interested to know how often this allele occurs in the global cucumber germplasm. Therefore, we performed an *in silico* screen on a collection of 115 recently resequenced cucumber accessions [8] for the presence and/or absence of the transposable element (TE) allele of *CsaMLO8*. We found evidence for the presence of the TE-allele, either homozygously or heterozygously, in at least 31 out of the 115 accessions (Table 1), indicating that this particular allele of *CsaMLO8* occurs quite often. For some accessions only a small number of reads indicating presence/absence of the TE allele was found, potentially due to a low read coverage at this locus. It is therefore possible that in some accessions now identified as homozygous for either the TE-allele or the WT allele of *CsaMLO8*, reads indicative of the other allele were missed due to low read coverage, so there might be some heterozygous accessions misidentified as being homozygous for one of the alleles.

As we found that the TE allele of *CsaMLO8* leads to PM resistance, it might have been selected for by cucumber breeders, by selecting for the most resistant plants. Interestingly one of the accessions found to have the TE-allele of *CsaMLO8* was PI 215589, a wild accession of *C. sativus var. hardwickii* collected in India in 1954. This indicates that the TE-allele of *CsaMLO8* does occur in the wild, and might have been introgressed in cultivated cucumber from PI 215589 or a related *hardwickii* accession.

## Conclusions

In this study we provide evidence for a role of *CsaMLO8* as a *S* gene for powdery mildew (PM) susceptibility. We show that complementation by *CsaMLO8* overexpression in *Slmlo1* mutant tomato background restores PM susceptibility. We also show that a mutant allele of *CsaMLO8* cloned from resistant cucumber fails to restore PM susceptibility. As *CsaMLO8* is located in the region where a QTL for hypocotyl specific resistance was detected, we determined *CsaMLO8* expression in different tissues of PM inoculated plants, and found that *CsaMLO8* was only transcriptionally upregulated in hypocotyl tissue. On this basis we conclude that the mutant allele of *CsaMLO8* is causal to the observed hypocotyl resistance towards PM in cucumber.

## Methods

### Plant materials and fungal strain

Two cucumber genotypes were used in this study: the PM susceptible cv. Sheila and an advanced breeding line, related to the resistant cv. Anaxo*,* homozygous for a recessively inherited QTL on chromosome 5 conferring hypocotyl resistance (*pm-h*).

Two tomato genotypes were used: PM susceptible cv. Moneymaker (MM), and a PM resistant breeding line *ol-2*, homozygous for a 19 bp deletion mutation in the coding sequence of *SlMLO1* [21].

Unless otherwise indicated, plants were grown under standard conditions in a closed greenhouse.

An isolate of *P. xanthii* (causing PM in cucumber) was obtained from infected cucumber plants in the greenhouse of a seeds company from The Netherlands and maintained on cv. Sheila in a greenhouse compartment at Wageningen University, The Netherlands. The species of the isolate was confirmed by sequencing of the ITS sequence from fungal DNA by primer pair 5′- CGTCAGAGAAGCCCCAACTC-3′ (ITS *P. xanthii* Forward) and 5′-AGCCAAGAGATCCGTTGTTG-3′ (ITS *P. xanthii* Reverse) (data not shown).

The Wageningen isolate of *Oidium neolycopersici* (tomato PM) was maintained on cv. MM as described [44].

### Cloning and sequencing of *CsaMLO8* CDS

Young leaves of cucumber cv. Sheila and the resistant breeding line were harvested and immediately frozen in liquid nitrogen. Total RNA was isolated by using the RNeasy Kit (Qiagen, Germany). Possible DNA contamination of RNA samples was removed by treatment with DNase I, Amp Grade (Invitrogen life technologies, U.S.A.). cDNA was synthesised using 2 μg of RNA samples with an iScript cDNA Synthesis Kit (Bio-Rad Laboratories, U.S.A.).

For amplification of *CsaMLO8* coding sequences, cDNA was amplified with primers 5′- caccCTGCCTCTCCACATGCATAA-3′ (Full length *CsaMLO8* Forward) and 5′-GCGCCCTGTACATGAAGAAC-3′ (Full length *CsaMLO8* Reverse). As template 50 ng cDNA was used in 50 μl reactions using 1 u *PfuUltra* II Fusion HS DNA polymerase (Agilent Technologies, U.S.A.), 1x reaction buffer, 1 mM dNTP and 200 nM of each primer. Cycling conditions were: 1 min. initial denaturation at 95 °C, followed by 40 cycles of 20 s. denaturation at 95 °C, 20 s. annealing at 60 °C and 2 min. extension at 72 °C. Reactions were finished by 3 min. incubation at 72 °C. PCR products were separated by gel electrophoresis in ethidium bromide stained agarose gels. Bands were cut out and purified using QIAquick Gel Extraction Kit (Qiagen, Germany). Purified products were cloned into Gateway-compatible vector pENTR D-TOPO (Invitrogen life technologies, U.S.A.) and transformed to chemically competent *Escherichia coli* strain One Shot TOP10. Presence of the right fragment was assessed by colony PCR using primers and conditions as above. Plasmids were recovered using the Qiaprep spin miniprep kit (Qiagen, Germany). Sequencing reactions were performed in triplicates using pUC/M13 forward and reverse sequencing primers (GATC Biotech, Germany).

### Complementation of tomato *ol-2* mutant with *CsaMLO8* WT and *CsaMLO8∆174*

Entry plasmids pENTR:*CsaMLO8* WT and pENTR:*CsaMLO8∆174*, obtained as described above, were transferred by Gateway LR cloning into binary vector pK7WG2, which harbours the constitutively active 35S Cauliflower Mosaic Virus promotor and the *nptII* marker gene for kanamycin resistance [45]. Recombinant plasmids were transformed to chemically competent *E. coli* strain dh5α. Positive recombinant bacterial colonies were screened by colony PCR using *CsaMLO8* specific primers as described above, and sequenced. Recombinant plasmids were recovered using the Qiaprep spin miniprep kit (Qiagen, Germany). pK7WG2:*CsaMLO8* WT and pK7WG2:*CsaMLO8∆174* binary vectors were transformed to electrocompetent cells of *Agrobacterium tumefaciens* strain AGL1-virG by electroporation [46].

Cotyledon explants of *ol-2* mutant tomato seedlings were transformed as previously described [25]. Obtained tomato transformants were assessed for presence of *CsaMLO8,* the *nptII* marker gene and the 35S CaMV promotor sequence by PCR with primers 5′- caccCTGCCTCTCCACATGCATAA-3′ (Full length *CsaMLO8* forward) and 5′-GCGCCCTGTACATGAAGAAC-3′ (Full length *CsaMLO8* reverse), 5′-GAAGGGACTGGCTGCTATTG-3′ (*nptII f*orward) and 5′-AATATCACGGGTAGCCAACG-3′ (*nptII r*everse), and 5′-TACAAAGGCGGCAACAAACG-3′ (35S forward) and 5′-AGCAAGCCTTGAATCGTCCA-3′ (35S reverse), with conditions as described above.

For each of the two transformations with a different construct, ten independent transgenic plants were selected, and were assessed for *CsaMLO8* expression by qRT-PCR using primer pair sequences specific for *CsaMLO8* 5′-GCGACGGCATTGAAGAACTG-3′ (Forward) and 5′-AGGAGACATGCCGTGAGTTG-3′ (Reverse). As housekeeping gene for normalization of *CsaMLO8* expression in tomato, *SlEF-α* was used, with primer pair 5′-ATTGGAAACGGATATGCCCCT-3′ (*SlEF-α* forward) and 5′-TCCTTACCTGAACGCCTGTCA-3′ (*SlEF-α* reverse). qRT-PCR was performed using the CFX96 Real-Time PCR machine (Bio-Rad Laboratories, U.S.A.). Each 10 μl reaction contained 300 nM of each primer, 1 μl (50 ng) cDNA template and 1 x iQ SYBR Green Supermix (Bio-Rad Laboratories, U.S.A.). Cycling conditions were an initial denaturation step of 95 °C for 3 min., followed by 40 cycles of 10 s. denaturation at 95 °C and 30 s. annealing and extension at 60 °C, finished by a melt cycle of 0.5 °C increment per 10 s. from 65 to 95 °C.

### Evaluation of PM resistance of *ol-2* tomato, overexpressing *CsaMLO8* WT or *CsaMLO8∆174*

Cuttings originating from ten individual transgenic plants per construct (two cuttings per plant) were inoculated with *O. neolycopersici.* Cuttings of an empty vector (EV) transformed *ol-2* plant and the susceptible cultivar Moneymaker (MM) were used as controls. A spore suspension was prepared by washing heavily infected leaves of cv. MM with water, and adjusting the spore concentration to 8 x 10^4^ conidiospores/ml . The spore suspension was evenly sprayed on the cuttings. Sixteen days after inoculation the disease severity was assessed by eye, and scored as either susceptible (sporulating powdery mildew colonies visible on leaves) or resistant (no powdery mildew symptoms at all). Additionally, leaf samples were taken for quantification of *O. neolycopersici* biomass. Infected leaves (the 2nd or 3rd leaf) were sampled for each cutting. Total plant and fungal DNA was extracted using the DNeasy Plant Kit (Qiagen, Germany). Isolated DNA was used for qPCR with primer pair 5′-CGCCAAAGACCTAACCAAAA-3′ (*Oidium* ITS forward) and 5′-AGCCAAGAGATCCGTTGTTG-3′ (*Oidium* ITS reverse), specific for the internal transcribed spacer (ITS) of *O. neolycopersici* ribosomal DNA, to quantify *O. neolycopersici* biomass, and with *SlEF-α* primers as described above for normalization. qPCR was performed using the CFX96 Real-Time PCR machine (Bio-Rad Laboratories, U.S.A.). Each 10 μl reaction contained 300 nM of each primer, 2 μl (20 ng) cDNA template and 1 x iQ SYBR Green Supermix (Bio-Rad Laboratories, U.S.A.). Cycling conditions were identical to those described above for quantification of *CsaMLO8* expression in transformed tomato.

### Amplification, sequencing and characterization of *CsaMLO8*-insertion

DNA was isolated from young leaves of cucumber cv. Sheila and the resistant breeding line, which were immediately frozen in liquid nitrogen after harvesting, using the DNeasy Plant Kit (Qiagen, Germany). DNA was amplified with primers 5′-AGCATTTTGCCATCCATACTTCA-3′ (*CsaMLO8* insertion region Forward) and 5′-CTGCAAGCACAGGATGAATGTC-3′ (*CsaMLO8* insertion region Reverse). As template 30 ng DNA was used in 25 μl reactions using 1.25 u DreamTaq DNA polymerase (Thermo Scientific, U.S.A.), 1x DreamTaq buffer, 0.8 mM dNTP and 200 nM of each primer. Cycling conditions were: 3 min. initial denaturation at 95 °C, followed by 35 cycles of 30 s. denaturation at 95 °C, 30 s. annealing at 57 °C and 2 min. extension at 72 °C. Reactions were finished by 5 min. incubation at 72 °C. PCR products were visualised by staining with GelRed and electrophoresis on agarose gels. PCR products were purified using Qiaquick PCR purification kit (Qiagen, Germany). Sequencing reactions were performed in duplo, using primers 5′-AGCATTTTGCCATCCATACTTCA-3′ (*CsaMLO8* insertion region Forward), 5′-ACGAAGAGCGAAACGAAGAA-3′ (*CsaMLO8* insertion sequencing Forward), 5′- GCTCCTGCCCAATTCAGACC-3′ (*CsaMLO8* insertion sequencing Reverse) and 5′-CTGCAAGCACAGGATGAATGTC-3′ (*CsaMLO8* insertion region Reverse) (GATC Biotech, Germany). Obtained sequences were aligned using CLC Genomics Workbench 7.5 software. The consensus sequence for the amplified region was extracted from the alignment. This consensus sequence was aligned to the genomic reference sequence of *CsaMLO8* to determine the exact location and sequence of the insertion.

A dot plot was constructed for the sequence of the insertion, using CLC Genomics Workbench 7.5 standard settings. The first and last 200 bp of the insertion sequence were extracted and aligned to each other to identify the length and sequence of the LTRs. The sequence of the insertion was scanned for open reading frames using CLC Genomics Workbench 7.5 standard settings, which gave no results.

### *In silico* mining of the cucumber reference genome for homologous TEs

The previously determined LTR sequence of the *CsaMLO8*-TE was used as query to perform a BLASTn search in the genome of the cucumber reference genome (Chinese long inbred line ‘9930’, v2 [7]) to identify putative homologous LTRs. The resulting output was stored as a tabular file. A python script described by Wolters et al. [32] was used to search for LTR matches within 20 kb from each other. Sequences with a length smaller than 20 kb flanked by two LTRs were considered as putative homologous TEs, and were extracted from the genome using the BEDtools suite [47]. The list of putative TEs was manually curated to remove sequences with two LTRs in opposite directions (two instances) and sequences with large (>100 bp) gaps (25 instances). In three instances, putative TEs were found to be nested (i.e., three LTRs were found to be within 20 kb of each other), in which cases the smaller putative TEs were discarded in favour of the bigger, nested model. Putative TEs were aligned to one another and to the *CsaMLO8*-TE using CLC Genomics Workbench 7.5 software, to determine sequence identity compared to the *CsaMLO8*-TE. Putative TEs were screened for open reading frames using CLC Genomics Workbench 7.5 standard settings. Putative TEs were used as query to perform tBLASTx searches to the REPbase database [48].

### *In silico* screening of resequenced lines for presence of *CsaMLO8*-TE allele

Reads of the resequencing project of 115 cucumber accessions by Qi et al. [8] were downloaded from the NCBI short read archive, accession SRA056480. By a simple Bash script, total reads were screened for the presence of 30 bp sequences comprised of:

1. The last 15 bp of *CsaMLO8* before the TE insertion and the first 15 bp of the TE insertion, in forward (5′- GCTCCATGTTATTATTGTTGATTTTATGGA-3′) or reverse (5′-TCCATAAAATCAACAATAATAACATGGAGC-3′) orientation;
2. The last 15 bp of the TE insertion and the first 15 bp of *CsaMLO8* after the TE insertion, in forward (5′-TATATTAATAATTATAACTCATATGGGATT-3′) or reverse (5′- AATCCCATATGAGTTATAATTATTAATATA-3′) orientation;
3. The 30 bp of *CsaMLO8* surrounding the TE insertion site, without TE sequence, in forward (5′- GCTCCATGTTATTATAACTCATATGGGATT-3′) or reverse (5′-AATCCCATATGAGTTATAATAACATGGAGC-3′) orientation.

The number of detected reads per accession with each of the six bait sequences was stored as a tabular file. The total number of reads indicating presence of the TE allele and the total number of reads indicating presence of the WT allele were summated, the genotype of the accessions was determined to be either homozygous TE-allele, homozygous WT-allele or heterozygous.

### *CsaMLO8* expression analysis PM-inoculated cucumber

PM susceptible and resistant cucumbers were grown in a climate chamber at 20 °C (day) and 16 °C (night), with a 16 h/8 h day/night cycle, and a relative humidity of 90 %. 18 days post seeding, plants were inoculated with a *P. xanthii* spore suspension by spray method, using inoculum that was obtained by washing heavily infected cucumber leaves with water. The inoculum was adjusted to a final concentration of 1.0 x 10^4^ conidia/ml. The spore suspension was evenly sprayed on leaves, cotyledons and hypocotyl of the seedlings. Prior to inoculation and at 4, 6, 8 and 24 h post inoculation (hpi), from three individual plants per time point hypocotyl, cotyledon and (first) true leaf samples were harvested separately, and were immediately frozen in liquid nitrogen.

Total RNA was isolated using the MagMAX-96 Total RNA Isolation kit (Ambion, U.S.A.). cDNA was synthesised using 1 μg of RNA samples with an iScript cDNA Synthesis Kit (Bio-Rad Laboratories, U.S.A.). Before use in qRT-PCR, cDNA samples were diluted 10-fold.

Quantitative real-time PCR was performed using a CFX96 Real-Time PCR machine (Bio-Rad Laboratories, U.S.A.). Primer pair sequences specific for *CsaMLO8* 5′-GCGACGGCATTGAAGAACTG-3′ (Forward) and 5′-AGGAGACATGCCGTGAGTTG-3′ (Reverse) were used to quantify *CsaMLO8* expression. Primer pairs specific for cucumber housekeeping genes *TIP41*, *CACS* and *EF-α*, as described by Warzybok et al. [49], were used for normalization of *CsaMLO8* expression. Each 10 μl reaction contained 300 nM of each primer, 1 μl (50 ng) cDNA template and 1 x iQ SYBR Green Supermix (Bio-Rad Laboratories, U.S.A.). Cycling conditions were an initial denaturation step of 95 °C for 3 min. followed by 40 cycles of 10 s. denaturation at 95 °C and 30 s. annealing and extension at 60 °C, finishing with a melt cycle of 0.5 °C increment per 10 s. from 65 to 95 °C.

Two technical replicates for each sample were tested. *CsaMLO8* expression of each sample was determined by the ∆∆C_t_ method [50], normalised by the geometric mean of the three housekeeping genes. Averages and standard errors of *CsaMLO8* transcript abundance were calculated over three biological replicates per tissue/time point combination, and statistical significance of differences in ∆∆C_t_ value between time points 4, 6, 8 and 24 hpi and 0 hpi were determined, using Student’s T-tests.

### Relative quantification of *CsaMLO8* transcript isoforms in resistant cucumber

cDNA samples of non-inoculated and inoculated (6 hpi) resistant cucumber tissues, obtained as described above, were used to quantify relative transcript abundance of the ∆174 and ∆72 splice isoforms. Quantitative real-time PCR was performed using a CFX96 Real-Time PCR machine (Bio-Rad Laboratories, U.S.A.). Four primer pairs were designed to specifically amplify one of the two *CsaMLO8* splice isoforms: 5′-CTCCTTAATTAATGCATTTCAGC-3′ (Forward) with 5′-CTTGTATGATAACCCCCATTGAG-3′ (Reverse) or 5′-TTCATTGTTGCACATCTTGC-3′ (Forward) with 5′-AAGCTGAAATGCATTAATTAAGG-3′(Reverse) for specific quantification of *CsaMLO8*∆174 and 5′-ATTCTATTGGGTGTTCCCGTC-3′ (Forward) with 5′-CTTGTATGATAACCCCCATTGAG-3′ (Reverse) or 5′-TTCATTGTTGCACATCTTGC-3′ (Forward) with 5′-GAACGACGGGAACACCCAAT-3′(Reverse) for specific quantification of *CsaMLO8*∆72. Primer pairs specific for cucumber housekeeping genes *TIP41*, *CACS* and *EF-α*, as described by Warzybok et al. [49], were used for normalization of *CsaMLO8* expression. Each 10 μl reaction contained 300 nM of each primer, 1 μl (50 ng) cDNA template and 1 x iQ SYBR Green Supermix (Bio-Rad Laboratories, U.S.A.). Cycling conditions were an initial denaturation step of 95 °C for 3 min. followed by 40 cycles of 10 s. denaturation at 95 °C and 30 s. annealing and extension at 60 °C, finishing with a melt cycle of 0.5 °C increment per 10 s. from 65 to 95 °C.

Two technical replicates for each sample were tested. *CsaMLO8* expression of each sample was determined by the ∆∆C_t_ method [50], normalised by the geometric mean of the three housekeeping genes. Averages and standard errors of *CsaMLO8* splice isoform abundance were calculated over three biological replicates per tissue, per tissue the average of the relative abundances calculated with the two different primer pairs per splice isoform was calculated.

## Additional files

Additional file 1:
**Full length alignment of** ***CsaMLO8*** **WT,** ***CsaMLO8∆72*** **and** ***CsaMLO8∆174*** **coding sequences.** (PDF 162 kb) Additional file 2:
**Multiple sequence alignment of MLO proteins encoded by clade V** ***MLO S*** **-genes from different species.** (PDF 287 kb) Additional file 3:
**Relative quantification of** ***CsaMLO8∆174*** **and** ***CsaMLO8∆72*** **transcript abundances by** **qRT-PCR** **on cDNA samples obtained from** **non-inoculated** **(A) or inoculated (B) cucumber tissue samples.** Fold changes were normalised relative to *CsaMLO8∆174* expression. Bars represent the average fold change over three independent biological replicates. Error bars indicate standard errors of the mean. (PDF 386 kb) Additional file 4:
**Photographs of 20 independent** ***ol-2*** **tomato plants transformed with either** ***CsaMLO8*** **WT or** ***CsaMLO8∆174.*** (PDF 343 kb) Additional file 5:
**Complete overview of putative LTRs and putative TEs homologous to the TE identified in** ***CsaMLO8.*** (XLSX 27 kb) Additional file 6:
**Multiple sequence alignment of the TE identified in** ***CsaMLO8*** **and putative homologous TEs.** (PDF 917 kb) Additional file 7:
**Full table of 115 resequenced accessions.** The amount of reads identified is given at the overlap between *CsaMLO8* and the start of the insertion in forward (TE start-F) and reverse (TE start-R) direction, at the overlap between the end of the insertion and *CsaMLO8* in forward (TE end-F) and reverse (TE end-R) direction, and at the site of the insertion with only *CsaMLO8* sequence in forward (WT-F) and reverse (WT-R) direction. (XLSX 22 kb)

## Abbreviations

PM: Powdery mildew
*MLO*: Mildew locus O
QTL: Quantitative trait locus
LTR: Long terminal repeat
NB-LRR: Nucleotide binding-leucine rich repeat
R-gene: Resistance gene
S-gene: Susceptibility gene
SNP: Single nucleotide polymorphism
WT: Wild type
TSD: Target site duplication
ORF: Open reading frame
TE: Transposable element
EV: Empty vector

## References

[1] Food and Agriculture Organization of the United Nations, Statistics Division. [http://faostat3.fao.org/] Accessed May 2015.

[2] Pérez-García A, Romero D, Fernández-Ortuño D, López-Ruiz F, De Vicente A, Torés JA (2009). The powdery mildew fungus *Podosphaera fusca* (synonym *Podosphaera xanthii*), a constant threat to cucurbits. Mol Plant Pathol.

[3] Block CC, Reitsma KR (2005). Powdery Mildew Resistance in the U. S. National Plant Germplasm System Cucumber Collection. Hortic Sci.

[4] Jahn M, Munger HM, McCreight JD (2002). Breeding cucurbit crops for powdery mildew resistance. The powdery mildews: a comprehensive treatise.

[5] Shanmugasundaram S, Williams PH, Peterson CE (1971). Inheritance of resistance to powdery mildew in cucumber. Phytopathology.

[6] Sitterly WR (1972). Breeding for disease resistance in cucurbits. Annu Rev Phytopathol.

[7] Huang S, Li R, Zhang Z, Li L, Gu X, Fan W, Lucas WJ, Wang X, Xie B, Ni P, Ren Y, Zhu H, Li J, Lin K, Jin W, Fei Z, Li G, Staub J, Kilian A, van der Vossen EAG, Wu Y, Guo J, He J, Jia Z, Ren Y, Tian G, Lu Y, Ruan J, Qian W, Wang M (2009). The genome of the cucumber, *Cucumis sativus* L. Nat Genet.

[8] Qi J, Liu X, Shen D, Miao H, Xie B, Li X, Zeng P, Wang S, Shang Y, Gu X, Du Y, Li Y, Lin T, Yuan J, Yang X, Chen J, Chen H, Xiong X, Huang K, Fei Z, Mao L, Tian L, Städler T, Renner SS, Kamoun S, Lucas WJ, Zhang Z, Huang S (2013). A genomic variation map provides insights into the genetic basis of cucumber domestication and diversity. Nat Genet.

[9] Wóycicki R, Witkowicz J, Gawroński P, Dąbrowska J, Lomsadze A, Pawełkowicz M, Siedlecka E, Yagi K, Pląder W, Seroczyńska A, Śmiech M, Gutman W, Niemirowicz-Szczytt K, Bartoszewski G, Tagashira N, Hoshi Y, Borodovsky M, Karpiński S, Malepszy S, Przybecki Z (2011). The genome sequence of the North-European cucumber (Cucumis sativus L.) unravels evolutionary adaptation mechanisms in plants. PLoS One.

[10] Jones JDG, Dangl JL (2006). The plant immune system. Nature.

[11] De Almeida EJ, Favery B, Engler G, Abad P (2005). Loss of susceptibility as an alternative for nematode resistance. Curr Opin Biotechnol.

[12] Van Schie CCN, Takken FLW (2014). Susceptibility genes 101: how to be a good host. Annu Rev Phytopathol.

[13] Pavan S, Jacobsen E, Visser RGF, Bai Y (2010). Loss of susceptibility as a novel breeding strategy for durable and broad-spectrum resistance. Mol Breed.

[14] Eckardt NA (2002). Plant disease susceptibility genes?. Plant Cell.

[15] Freisleben R, Metzger I (1942). Über die Auffindung einer mehltauresistenten Mutante nach Röntgenbestrahlung einer anfälligen reinen Linie von Sommergerste. Naturwissenschaften.

[16] Jorgensen JH (1992). Discovery, characterization and exploitation of Mlo powdery mildew resistance in barley. Euphytica.

[17] Büschges R, Hollricher K, Panstruga R, Simons G, Wolter M, Frijters A, van Daelen R, van der Lee T, Diergaarde P, Groenendijk J, Töpsch S, Vos P, Salamini F, Schulze-Lefert P (1997). The barley *Mlo* gene: a novel control element of plant pathogen resistance. Cell.

[18] Devoto A, Piffanelli P, Nilsson M, Wallin E, Panstruga R, von Heijne G, Schulze-Lefert P (1999). Topology, subcellular localization, and sequence diversity of the Mlo family in plants. J Biol Chem.

[19] Devoto A, Hartmann HA, Piffanelli P, Elliott C, Simmons C, Taramino G, Goh C-S, Cohen FE, Emerson BC, Schulze-Lefert P, Panstruga R (2003). Molecular phylogeny and evolution of the plant-specific seven-transmembrane MLO family. J Mol Evol.

[20] Consonni C, Humphry ME, Hartmann HA, Livaja M, Durner J, Westphal L, Vogel J, Lipka V, Kemmerling B, Schulze-Lefert P, Somerville SC, Panstruga R (2006). Conserved requirement for a plant host cell protein in powdery mildew pathogenesis. Nat Genet.

[21] Bai Y, Pavan S, Zheng Z, Zappel NF, Reinstädler A, Lotti C, De Giovanni C, Ricciardi L, Lindhout P, Visser R, Theres K, Panstruga R (2008). Naturally occurring broad-spectrum powdery mildew resistance in a Central American tomato accession is caused by loss of *Mlo* function. Mol Plant-Microbe Interact.

[22] Humphry M, Reinstädler A, Ivanov S, Bisseling T, Panstruga R (2011). Durable broad-spectrum powdery mildew resistance in pea er1 plants is conferred by natural loss-of-function mutations in *PsMLO1*. Mol Plant Pathol.

[23] Zheng Z, Nonomura T, Appiano M, Pavan S, Matsuda Y, Toyoda H, Wolters A-MA, Visser RGF, Bai Y (2013). Loss of function in *Mlo* orthologs reduces susceptibility of pepper and tomato to powdery mildew disease caused by *Leveillula taurica*. PLoS One.

[24] Wang Y, Cheng X, Shan Q, Zhang Y, Liu J, Gao C, Qiu J-L (2014). Simultaneous editing of three homoeoalleles in hexaploid bread wheat confers heritable resistance to powdery mildew. Nat Biotechnol.

[25] Appiano M, Pavan S, Catalano D, Zheng Z, Bracuto V, Lotti C, Visser RGF, Ricciardi L, Bai Y (2015). Identification of candidate *MLO* powdery mildew susceptibility genes in cultivated Solanaceae and functional characterization of tobacco *NtMLO1*. Transgenic Res.

[26] Feechan A, Jermakow AM, Torregrosa L, Panstruga R, Dry IB (2008). Identification of grapevine *MLO* gene candidates involved in susceptibility to powdery mildew. Funct Plant Biol.

[27] Jiwan D, Roalson EH, Main D, Dhingra A (2013). Antisense expression of peach mildew resistance locus O (*PpMlo1*) gene confers cross-species resistance to powdery mildew in *Fragaria x ananassa*. Transgenic Res.

[28] Schouten HJ, Krauskopf J, Visser RGF, Bai Y. Identification of candidate genes required for susceptibility to powdery or downy mildew in cucumber. Euphytica. 2014;200:475–486.

[29] He X, Li Y, Pandey S, Yandell BS, Pathak M, Weng Y (2013). QTL mapping of powdery mildew resistance in WI 2757 cucumber (Cucumis sativus L.). Theor Appl Genet.

[30] Tusnady GE, Simon I (2001). The HMMTOP transmembrane topology prediction server. Bioinformatics.

[31] Wicker T, Sabot F, Hua-Van A, Bennetzen JL, Capy P, Chalhoub B, Flavell A, Leroy P, Morgante M, Panaud O, Paux E, SanMiguel P, Schulman AH (2007). A unified classification system for eukaryotic transposable elements. Nat Rev Genet.

[32] Wolters PJ, Schouten HJ, Si-Ammour A, Baldi P. Genomic characterisation of the “Wijcik” mutation. Wageningen, The Netherlands: Wageningen University; 2014

[33] Kumar A, Bennetzen JL (1999). Plant retrotransposons. Annu Rev Genet.

[34] Neumann P, Požárková D, Macas J (2003). Highly abundant pea LTR retrotransposon *Ogre* is constitutively transcribed and partially spliced. Plant Mol Biol.

[35] Piffanelli P, Zhou F, Casais C, Orme J, Jarosch B, Schaffrath U, Collins NC, Panstruga R, Schulze-Lefert P (2002). The barley MLO modulator of defense and cell death is responsive to biotic and abiotic stress stimuli. Plant Physiol.

[36] Pessina S, Pavan S, Catalano D, Gallotta A, Visser RGF, Bai Y, Malnoy M, Schouten HJ (2014). Characterization of the *MLO* gene family in Rosaceae and gene expression analysis in *Malus domestica*. BMC Genomics.

[37] Diergaarde PJ, van Enckevort LJG, Posthuma KI, Prins MW (2012). Powdery Mildew Resistance Providing Genes in *Cucumis Melo*.

[38] Elliott C, Uller JM, Miklis M, Bhat RA, Schulze-lefert P, Panstruga R (2005). Conserved extracellular cysteine residues and cytoplasmic loop – loop interplay are required for functionality of the heptahelical MLO protein. J Biol Chem.

[39] Bennetzen JL (2000). Transposable element contributions to plant gene and genome evolution. Plant Mol Biol.

[40] Tenaillon MI, Hollister JD, Gaut BS (2010). A triptych of the evolution of plant transposable elements. Trends Plant Sci.

[41] Oliver KR, McComb JA, Greene WK (2013). Transposable elements: Powerful contributors to angiosperm evolution and diversity. Genome Biol Evol.

[42] Nie J, He H, Peng J, Yang X, Bie B, Zhao J, Wang Y, Si L, Pan J-S, Cai R (2015). Identification and fine mapping of *pm5.1*: a recessive gene for powdery mildew resistance in cucumber (*Cucumis sativus* L.). Mol Breed.

[43] Diergaarde PJ, van Enckevort LJG, Posthuma KI, Prins MW (2013). Powdery Mildew Resistance Providing Genes in Cucumis Sativus.

[44] Bai Y, van der Hulst R, Bonnema G, Marcel TC, Meijer-Dekens F, Niks RE, Lindhout P (2005). Tomato defense to *Oidium neolycopersici*: dominant *Ol* genes confer isolate-dependent resistance via a different mechanism than recessive *ol-2*. Mol Plant-Microbe Interact.

[45] Karimi M, Inzé D, Depicker A (2002). GATEWAY vectors for *Agrobacterium*-mediated plant transformation. Trends Plant Sci.

[46] Lazo GR, Stein PA, Ludwig RA (1991). A DNA transformation-competent *Arabidopsis* genomic library in *Agrobacterium*. Nat Biotechnol.

[47] Quinlan AR, Hall IM (2010). BEDTools: A flexible suite of utilities for comparing genomic features. Bioinformatics.

[48] Jurka J, Kapitonov VV, Pavlicek A, Klonowski P, Kohany O, Walichiewicz J (2005). Repbase Update, a database of eukaryotic repetitive elements. Cytogenet Genome Res.

[49] Warzybok A, Migocka M (2013). Reliable reference genes for normalization of gene expression in cucumber grown under different nitrogen nutrition. PLoS One.

[50] Pfaffl MW (2001). A new mathematical model for relative quantification in real-time RT-PCR. Nucleic Acids Res.

