# Supplementary material for: A transposable element insertion in the susceptibility gene CsaMLO8 results in hypocotyl resistance to powdery mildew in cucumber
Source: BMC Plant Biol. 2015 Oct 9;15:243. doi: 10.1186/s12870-015-0635-x (PMC4600303; doi:10.1186/s12870-015-0635-x)
Supplement: Additional file 1: — Full length alignment of CsaMLO8 WT, CsaMLO8∆72 and CsaMLO8∆174 coding sequences. (PDF 162 kb) [file 12870_2015_635_MOESM1_ESM.pdf]

```
                                      20                      40                      60                      80
CsaMLO8 WT    ATGGCTGAAT GTGGAACAGA GCAGCGTACT TTGGAAGATA CCTCAACTTG GGCTGTTGCG GTTGTTTGTT TTTTCTTGGT  80
CsaMLO8Δ72    ATGGCTGAAT GTGGAACAGA GCAGCGTACT TTGGAAGATA CCTCAACTTG GGCTGTTGCG GTTGTTTGTT TTTTCTTGGT  80
CsaMLO8Δ174   ATGGCTGAAT GTGGAACAGA GCAGCGTACT TTGGAAGATA CCTCAACTTG GGCTGTTGCG GTTGTTTGTT TTTTCTTGGT  80

                                     100                     120                     140                     160
CsaMLO8 WT    TGTTATTTCA ATCTTCATTG AACATGTCAT TCACCTCACT GGAAAGTGGC TGGAGAAAAG GCACAAGCCA GCTCTTGTTG  160
CsaMLO8Δ72    TGTTATTTCA ATCTTCATTG AACATGTCAT TCACCTCACT GGAAAGTGGC TGGAGAAAAG GCACAAGCCA GCTCTTGTTG  160
CsaMLO8Δ174   TGTTATTTCA ATCTTCATTG AACATGTCAT TCACCTCACT GGAAAGTGGC TGGAGAAAAG GCACAAGCCA GCTCTTGTTG  160

                                     180                     200                     220                     240
CsaMLO8 WT    AAGCTCTAGA AAAGGTTAAA GCAGAGCTTA TGCTATTGGG ATTCATATCC CTACTTCTAA CGATAGGCCA AGATGCTGTC  240
CsaMLO8Δ72    AAGCTCTAGA AAAGGTTAAA GCAGAGCTTA TGCTATTGGG ATTCATATCC CTACTTCTAA CGATAGGCCA AGATGCTGTC  240
CsaMLO8Δ174   AAGCTCTAGA AAAGGTTAAA GCAGAGCTTA TGCTATTGGG ATTCATATCC CTACTTCTAA CGATAGGCCA AGATGCTGTC  240

                                     260                     280                     300                     320
CsaMLO8 WT    ACTCAAATTT GTGTTTCGAA AGAGCTTGCA GCAACTTGGC TTCCCTGTGC AGCAAGAGCT AAAACAGGAG TAAAAGTTGC  320
CsaMLO8Δ72    ACTCAAATTT GTGTTTCGAA AGAGCTTGCA GCAACTTGGC TTCCCTGTGC AGCAAGAGCT AAAACAGGAG TAAAAGTTGC  320
CsaMLO8Δ174   ACTCAAATTT GTGTTTCGAA AGAGCTTGCA GCAACTTGGC TTCCCTGTGC AGCAAGAGCT AAAACAGGAG TAAAAGTTGC  320

                                     340                     360                     380                     400
CsaMLO8 WT    GAAGAACAGT CGTCTTAGAC TTCTTGAATT TTTAGATCCT GACTATGGTT CGAGGCGTAT TTTAGCCTCG AAAGGAGATG  400
CsaMLO8Δ72    GAAGAACAGT CGTCTTAGAC TTCTTGAATT TTTAGATCCT GACTATGGTT CGAGGCGTAT TTTAGCCTCG AAAGGAGATG  400
CsaMLO8Δ174   GAAGAACAGT CGTCTTAGAC TTCTTGAATT TTTAGATCCT GACTATGGTT CGAGGCGTAT TTTAGCCTCG AAAGGAGATG  400

                                     420                     440                     460                     480
CsaMLO8 WT    ATGCATGCGC TAAGAGGGGC CAACTCGCTT TCGTGTCGGC ATATGGAATC CATCAGCTCC ATATTTTCAT CTTCGTATTG  480
CsaMLO8Δ72    ATGCATGCGC TAAGAGGGGC CAACTCGCTT TCGTGTCGGC ATATGGAATC CATCAGCTCC ATATTTTCAT CTTCGTATTG  480
CsaMLO8Δ174   ATGCATGCGC TAAGAGGGGC CAACTCGCTT TCGTGTCGGC ATATGGAATC CATCAGCTCC ATATTTTCAT CTTCGTATTG  480

                                     500                     520                     540                     560
CsaMLO8 WT    GCTGTCTTCC ATGTCCTGTA CTGCATCATA ACTTTGGCTT TTGGCAGAAC AAAGATGAGC AAATGGAAGG CCTGGGAGGA  560
CsaMLO8Δ72    GCTGTCTTCC ATGTCCTATA CTGCATCATA ACTTTGGCTT TTGGCAGAAC AAAGATGAGC AAATGGAAGG CCTGGGAGGA  560
CsaMLO8Δ174   GCTGTCTTCC ATGTCCTATA CTGCATCATA ACTTTGGCTT TTGGCAGAAC AAAGATGAGC AAATGGAAGG CCTGGGAGGA  560

                                     580                     600                     620                     640
CsaMLO8 WT    TGAAACCAAG ACAATTGAAT ACCAGTACTA TAATGATCCA GCAAGATTTA GATTTGCTAG AGATACTACG TTTGGACGCC  640
CsaMLO8Δ72    TGAAACCAAG ACAATTGAAT ACCAGTACTA TAATGATCCA GCAAGATTTA GATTTGCTAG AGATACTACG TTTGGACGCC  640
CsaMLO8Δ174   TGAAACCAAG ACAATTGAAT ACCAGTACTA TAATGATCCA GCAAGATTTA GATTTGCTAG AGATACTACG TTTGGACGCC  640

                                     660                     680                     700                     720
CsaMLO8 WT    GACACTTGAG CTTCTGGAGT CGTACACCAA TTTCCCTCTG GATTGTTTGT TTCTTCAGAC AGTTCTTTGG ATCAGTTACC  720
CsaMLO8Δ72    GACACTTGAG CTTCTGGAGT CGTACACCAA TTTCCCTCTG GATTGTTTGT TTCTTCAGAC AATTCTTTGG ATCAGTTACC  720
CsaMLO8Δ174   GACACTTGAG CTTCTGGAGT CGTACACCAA TTTCCCTCTG GATTGTTTGT TTCTTCAGAC AATTCTTTGG ATCAGTTACC  720

                                     740                     760                     780                     800
CsaMLO8 WT    AAGGTTGATT ACATGACACT GAGACATGGA TTCATTGTTG CACATCTTGC ACCCGGAAGT GAAGTAAAAT TTGATTTCCA  800
CsaMLO8Δ72    AAGGTTGATT ACATGACACT GAGACATGGA TTCATCGTTG CACATCTTGC ACCCGGAAGT GAAGTAAAAT TTGATTTCCA  800
CsaMLO8Δ174   AAGGTTGATT ACATGACACT GAGACATGGA TTCATCGTTG CACATCTTGC ACCCGGAAGT GAAGTAAAAT TTGATTTCCA  800

                                     820                     840                     860                     880
CsaMLO8 WT    CAAATACATT AGCAGATCTC TGGAAGACGA CTTTAAAGTT GTTGTGGGGA TTAGTCCCGC AATGTGGCTA TTTGCTGTTC  880
CsaMLO8Δ72    CAAATACATT AGCAGATCTC TGGAAGACGA CTTTAAAGTT GTTGTGGGGA TTAGTCCCGC AATGTGGCTA TTTGCTGTTC  880
CsaMLO8Δ174   CAAATACATT AGCAGATCTC TGGAAGACGA CTTTAAAGTT GTTGTGGGGA TTAGTCCCGC AATGTGGCTA TTTGCTGTTC  880

                                     900                     920                     940                     960
CsaMLO8 WT    TCTTCATCCT AACCAATACA AATGGGTGGT ATTCATATCT ATGGCTGCCT TTCATCTCCT TAATTATAAT TCTATTGGTG  960
CsaMLO8Δ72    TCTTCATCCT AACCAATACA AATGGGTGGT ATTCATATCT ATGGCTGCCT TTCATCTCCT TAATTATAAT TCTATTG---  957
CsaMLO8Δ174   TCTTCATCCT AACCAATACA AATGGGTGGT ATTCATATCT ATGGCTGCCT TTCATCTCCT TAATT----- ----------  945

                                     980                   1,000                   1,020                   1,040
CsaMLO8 WT    GGAACAAAGC TCCATGTTAT TATAACTCAT ATGGGATTGA CAATTCAAGA AAGGGGTCAT GTTGTGAAGG GTGTTCCCGT  1040
CsaMLO8Δ72    ---------- ---------- ---------- ---------- ---------- ---------- ---------G GTGTTCCGGT  968
CsaMLO8Δ174   ---------- ---------- ---------- ---------- ---------- ---------- ---------- ----------  945

                                   1,060                   1,080                   1,100                   1,120
CsaMLO8 WT    CGTTCAGCCT CGGGATGACC TGTTTTGGTT TGGACGTCCA CAACTTATTC TCTTCCTGAT CCACTTTGTT CTCTTTATGA  1120
CsaMLO8Δ72    CGTTCAGCCT CGGGATGACC TGTTTTGGTT TGGACGTCCA CAACTTATTC TCTTCCTGAT CCACTTTGTT CTCTTTATGA  1048
CsaMLO8Δ174   ---------- ---------- ---------- ---------- ---------- ---------- ---------- ---------A  946

                                   1,140                   1,160                   1,180                   1,200
CsaMLO8 WT    ATGCATTTCA GCTTGCCTTC TTTGCTTGGA CCACATATGC ATTTAAGTGG ATGGGTTGTT TCCATCAGCG AGTTGAAGAT  1200
CsaMLO8Δ72    ATGCATTTCA GCTTGCCTTC TTTGCTTGGA CCACTTATGC ATTTAAGTGG ATGGGTTGTT TCCATCAGCG AGTTGAAGAT  1128
CsaMLO8Δ174   ATGCATTTCA GCTTGCCTTC TTTGCTTGGA CCACTTATGC ATTTAAGTGG ATGGGTTGTT TCCATCAGCG AGTTGAAGAT  1026

                                   1,220                   1,240                   1,260                   1,280
CsaMLO8 WT    ATTGTCATCA GACTCTCAAT GGGGGTTATC ATACAAGTTC TCTGCAGTTA TGTCACACTC CCACTCTATG CTTTGGTTAC  1280
CsaMLO8Δ72    ATTGTCATCA GACTCTCAAT GGGGGTTATC ATACAAGTTC TCTGCAGTTA TGTCACACTC CCACTCTATG CTTTGGTTAC  1208
CsaMLO8Δ174   ATTGTCATCA GACTCTCAAT GGGGGTTATC ATACAAGTTC TCTGCAGTTA TGTCACACTC CCACTCTATG CTTTGGTTAC  1106

                                   1,300                   1,320                   1,340                   1,360
CsaMLO8 WT    TCAGATGGGC TCTAACATGA GACCAACCAT TTTCAACGAC CGAGTGGCGA CGGCATTGAA GAACTGGCAC CACTCAGCCA  1360
CsaMLO8Δ72    TCAGATGGGC TCTAACATGA GACCAACCAT TTTCAACGAC CGAGTGGCAA CGGCATTGAA GAACTGGCAC CACTCCGCCA  1288
CsaMLO8Δ174   TCAGATGGGC TCTAACATGA GACCAACCAT TTTCAACGAC CGAGTGGCAA CGGCATTGAA GAACTGGCAC CACTCCGCCA  1186

                                   1,380                   1,400                   1,420                   1,440
CsaMLO8 WT    AGAAGAACAT GAAGCAGCAC CGCAACCCAG ACAGTACCTC ACCATTCTCA AGCAGGCCAG CTACTCCAAC TCACGGCATG  1440
CsaMLO8Δ72    AGAAGAACAT GAAGCAGCAC CGCAACCCAG ACAGTACCTC ACCATTCTCA AGCAGGCCAG CTACTCCAAC TCACGGCATG  1368
CsaMLO8Δ174   AGAAGAACAT GAAGCAGCAC CGCAACCCAG ACAGTACCTC ACCATTCTCA AGCAGGCCAG CTACTCCAAC TCACGGCATG  1266
```

```
                1,460                 1,480                 1,500                 1,520
                    |                     |                     |                     |
CsaMLO8 WT    TCTCCTATTC ACCTTCTGCA CAAACATCAG CATGGCAGCA CATCTCCCAG GCTATCCGAT GCCGAACCCG ATCGTTGGGA 1520
CsaMLO8Δ72    TCTCCTATTC ACCTTCTGCA CAAACATCAG CATGGCAGCA CATCTCCCAG GCTATCCGAT GCCGAACCCG ATCGTTGGGA 1448
CsaMLO8Δ174   TCTCCTATTC ACCTTCTGCA CAAACATCAG CATGGCAGCA CATCTCCCAG GCTATCCGAT GCCGAACCCG ATCGTTGGGA 1346

                1,540                 1,560                 1,580                 1,600
                    |                     |                     |                     |
CsaMLO8 WT    AGAGTTGCCT CCTTCTTCAC ACCATAGTAG AGCCCCCCAT CATGATAATC ATCAAGATCA ACAAGAACAA TCTGAGACAA 1600
CsaMLO8Δ72    AGAGTTGCCT CCTTCTTCAC ACCATAGTAG AGCCCCCCAT CATGATAATC ATCAAGATCA ACAAGAACAA TCTGAGACAA 1528
CsaMLO8Δ174   AGAGTTGCCT CCTTCTTCAC ACCATAGTAG AGCCCCCCAT CATGATAATC ATCAAGATCA ACAAGAACAA TCTGAGACAA 1426

                1,620                 1,640                 1,660                 1,680
                    |                     |                     |                     |
CsaMLO8 WT    TAATTAGAGA ACAGGAGATG ACAGTTCAAG GACCAAGTTC AAGTGAAACC GGTTCCATAA CACGTCCTGC TCGCCCTCAT 1680
CsaMLO8Δ72    TAATTAGAGA ACAGGAGATG ACAGTTCAAG GACCAAGTTC AAGTGAAACC GGTTCCATAA CACGTCCTGC TCGCCCTCAT 1608
CsaMLO8Δ174   TAATTAGAGA ACAGGAGATG ACAGTTCAAG GACCAAGTTC AAGTGAAACC GGTTCCATAA CACGTCCTGC TCGCCCTCAT 1506

                1,700                 1,720
                    |                     |
CsaMLO8 WT    CAGGAAATCA CTAGGACTCC ATCAGACTTC TCATTTGCCA AATGA 1725
CsaMLO8Δ72    CAGGAAATCA CTAGGACTCC ATCAGACTTC TCATTTGCCA AATGA 1653
CsaMLO8Δ174   CAGGAAATCA CTAGGACTCC ATCAGACTTC TCATTTGCCA AATGA 1551
```
