# Supplementary figures and images for: A transposable element insertion in the susceptibility gene CsaMLO8 results in hypocotyl resistance to powdery mildew in cucumber
Source: BMC Plant Biol. 2015 Oct 9;15:243. doi: 10.1186/s12870-015-0635-x (PMC4600303; doi:10.1186/s12870-015-0635-x)

**A**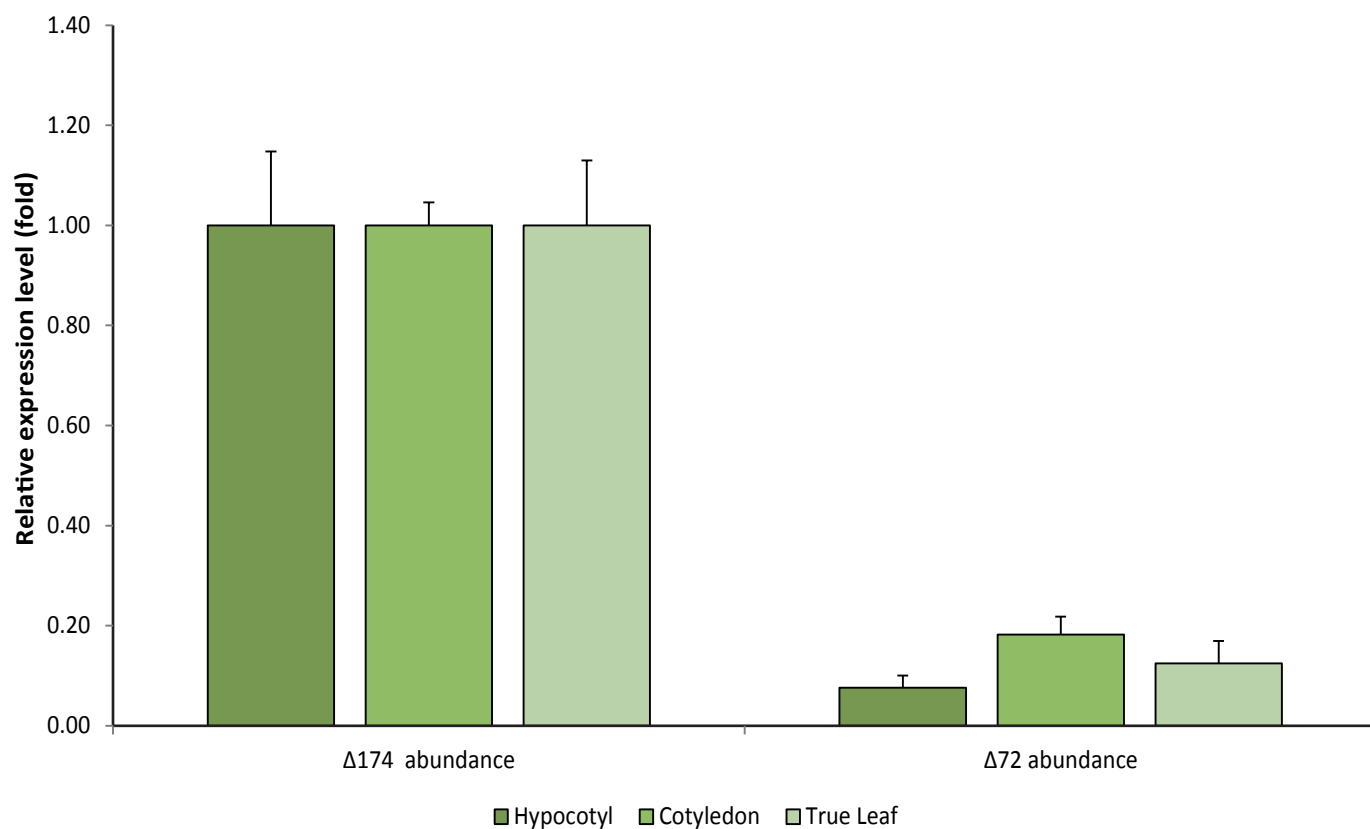**B**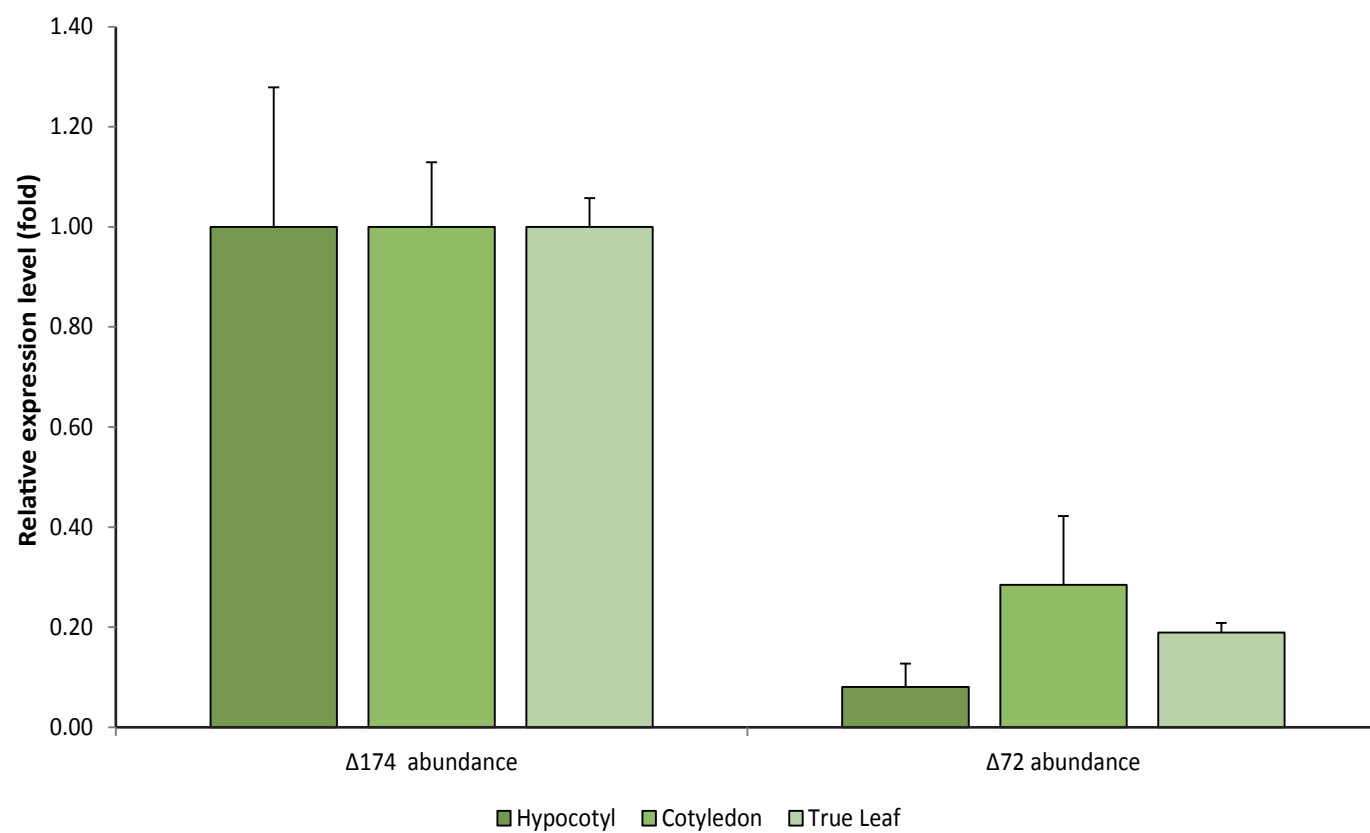

Supplement: Additional file 3: — Relative quantification of CsaMLO8∆174 and CsaMLO8∆72 transcript abundances by qRT-PCR on cDNA samples obtained from non-inoculated (A) or inoculated (B) cucumber tissue samples. Fold changes were normalised relative to CsaMLO8∆174 expression. Bars represent the average fold change over three independent biological replicates. Error bars indicate standard errors of the mean. (PDF 386 kb) [file 12870_2015_635_MOESM3_ESM.pdf]

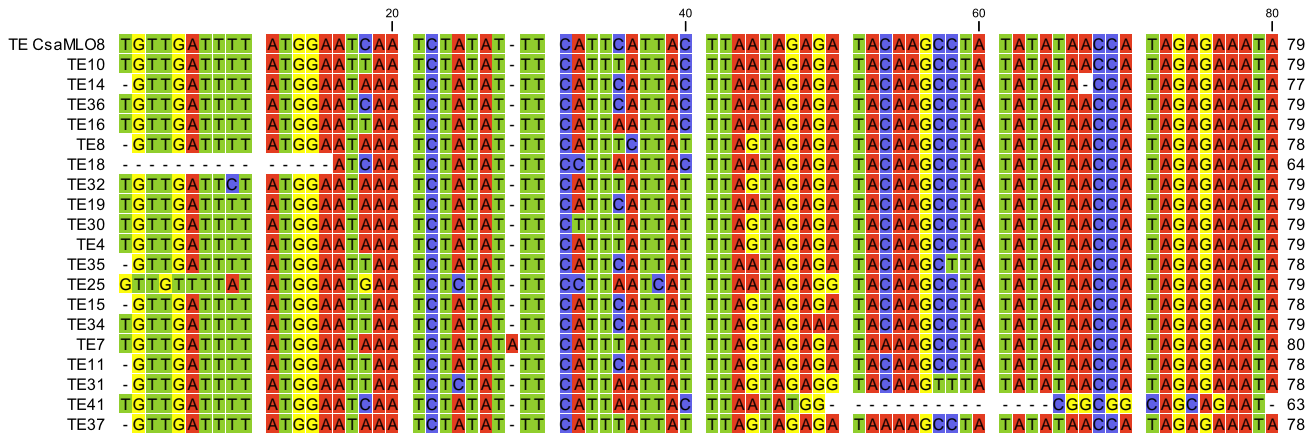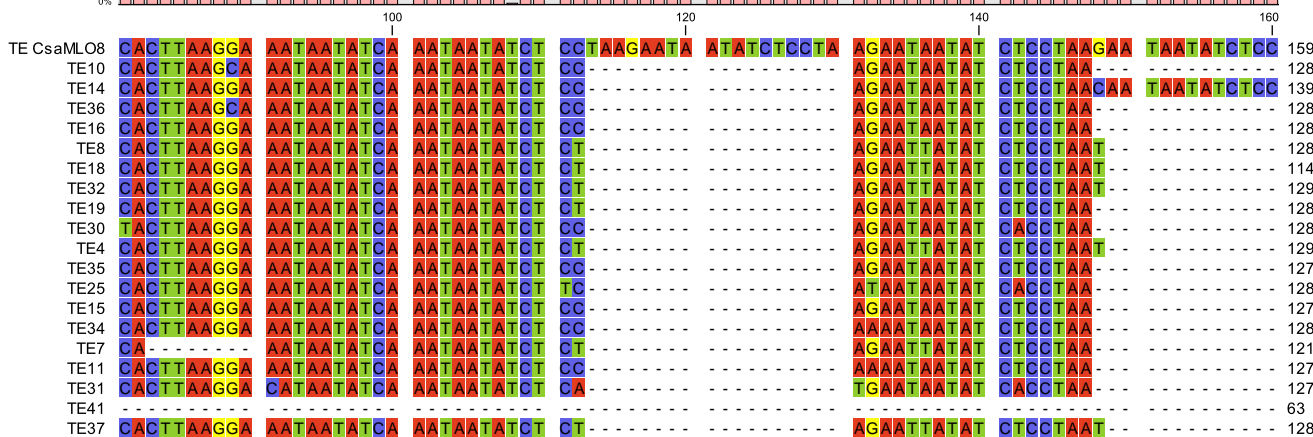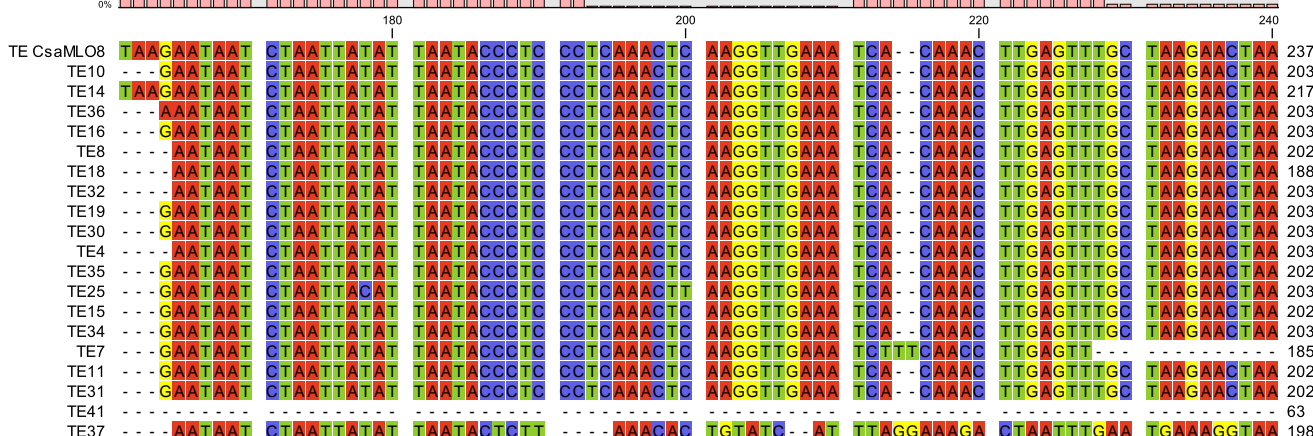

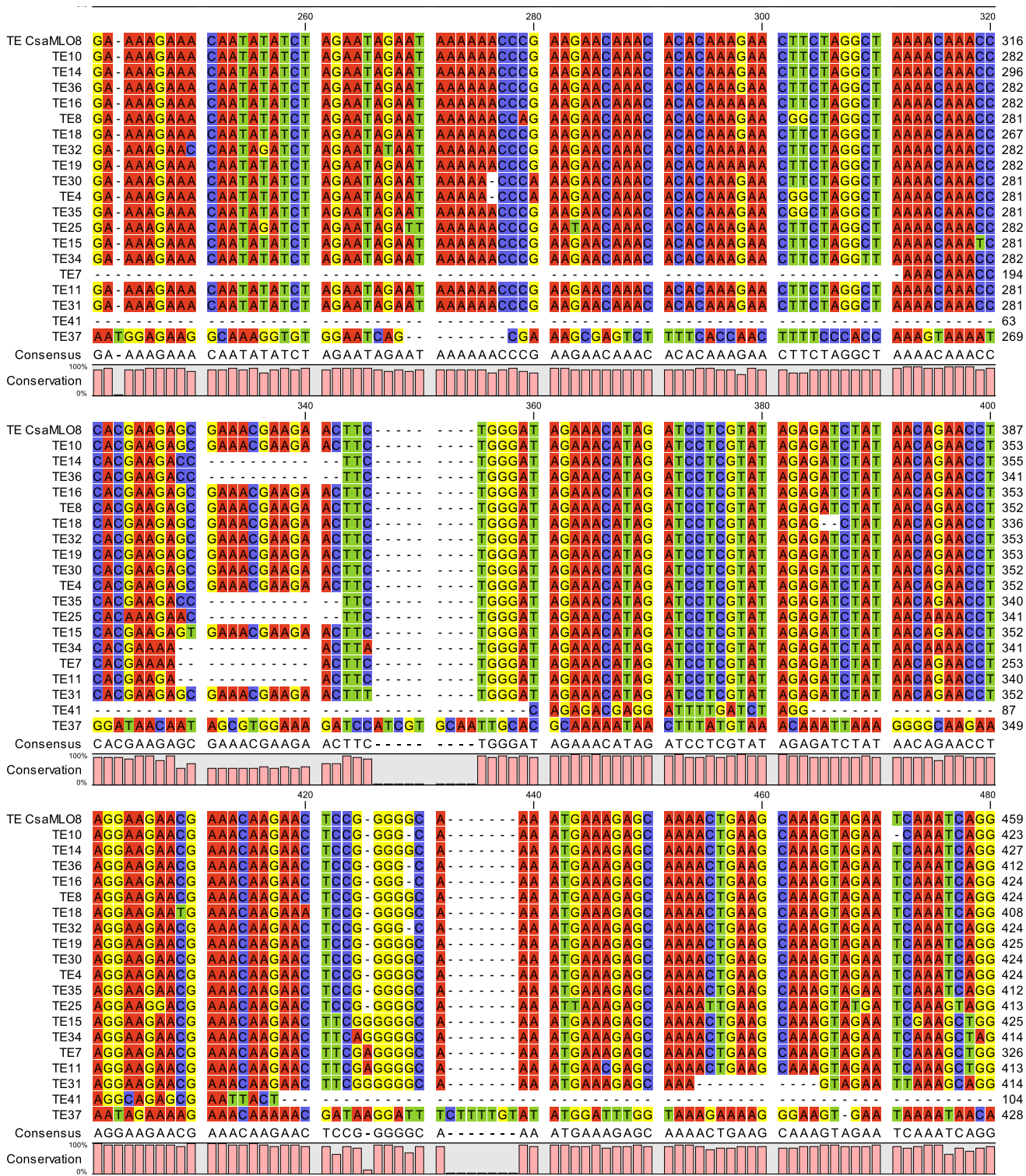



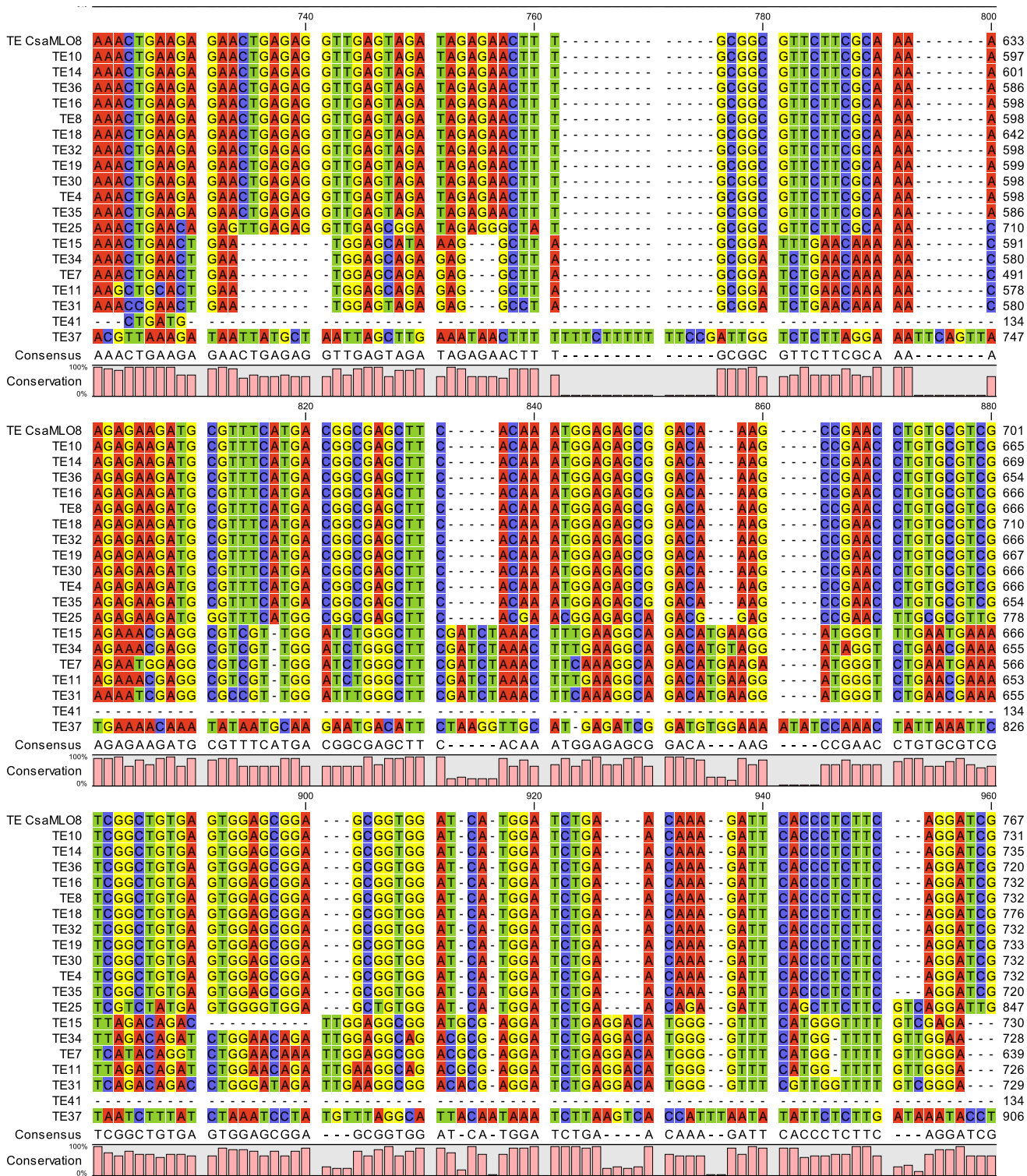

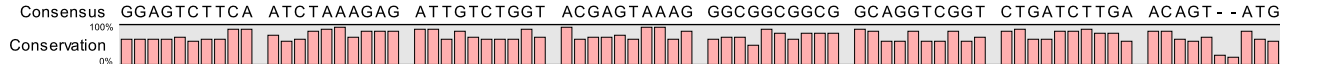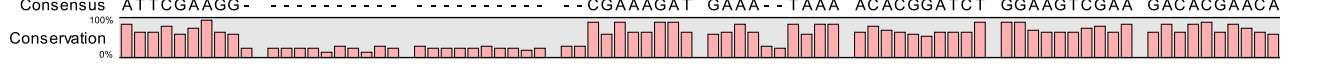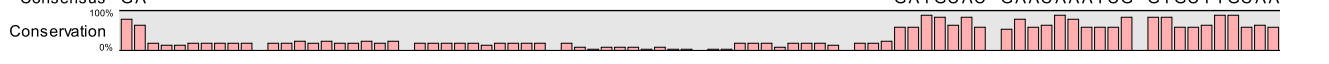



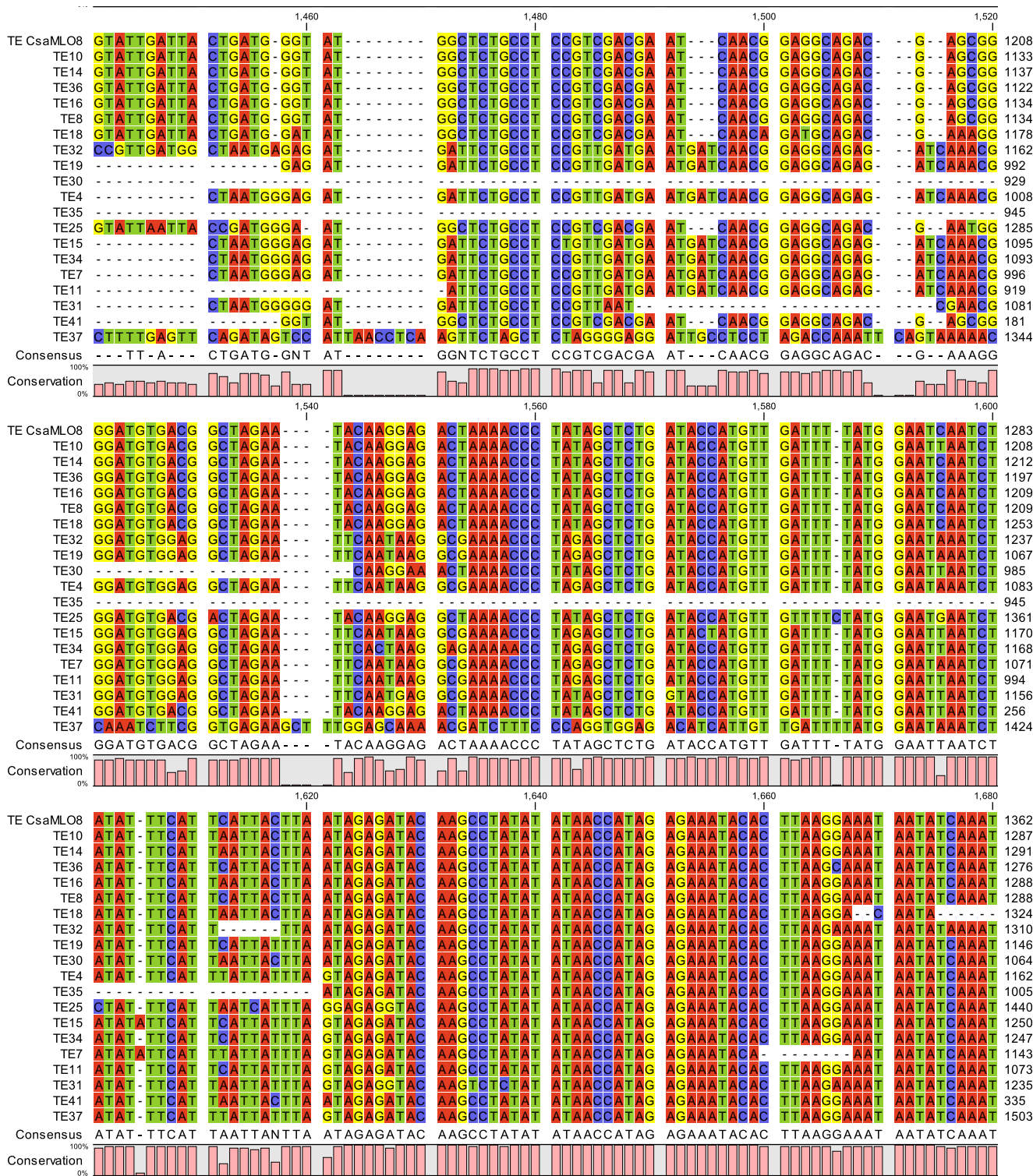

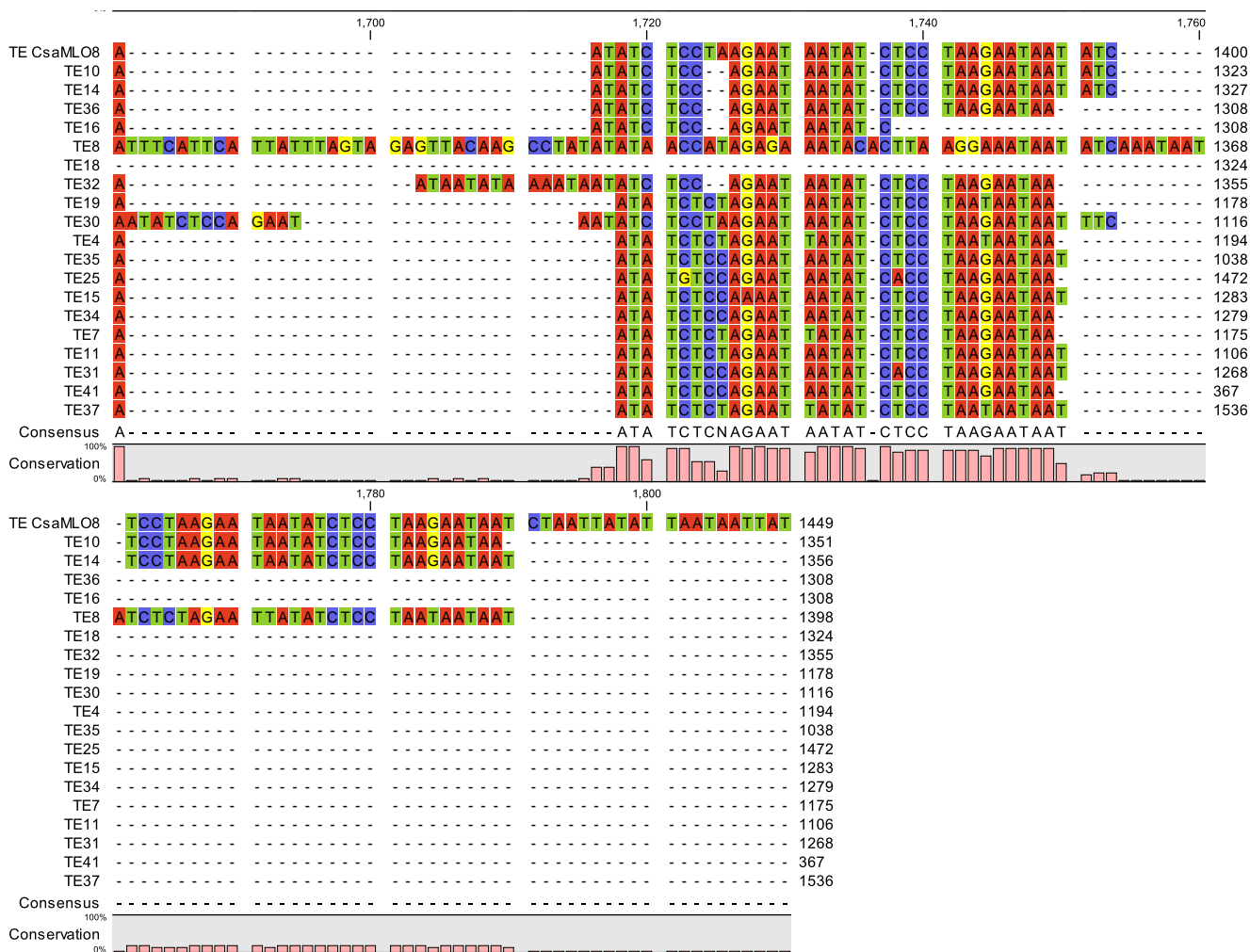

Supplement: Additional file 6: — Multiple sequence alignment of the TE identified in CsaMLO8 and putative homologous TEs. (PDF 917 kb) [file 12870_2015_635_MOESM6_ESM.pdf]
