# Supplementary material for: A transposable element insertion in the susceptibility gene CsaMLO8 results in hypocotyl resistance to powdery mildew in cucumber
Source: BMC Plant Biol. 2015 Oct 9;15:243. doi: 10.1186/s12870-015-0635-x (PMC4600303; doi:10.1186/s12870-015-0635-x)
Supplement: Additional file 4: — Photographs of 20 independent ol-2 tomato plants transformed with either CsaMLO8 WT or CsaMLO8∆174. (PDF 343 kb) [file 12870_2015_635_MOESM4_ESM.pdf]

## *ol-2* 35S::*CsaMLO8* WT transformants:

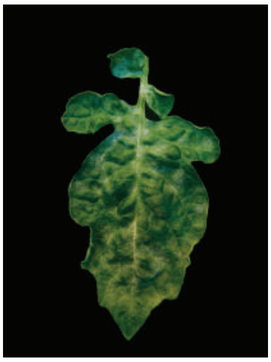
35S::CsaMLO8 WT - A

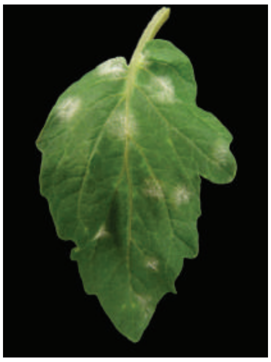
35S::CsaMLO8 WT - B

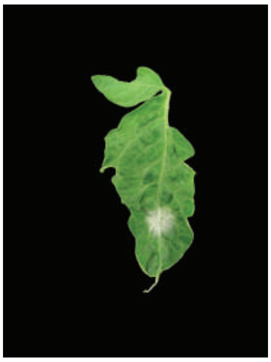
35S::CsaMLO8 WT - C

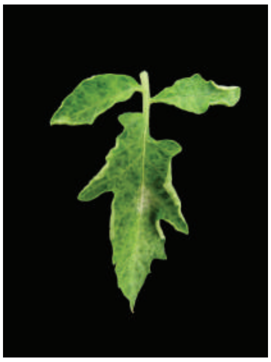
35S::CsaMLO8 WT - D

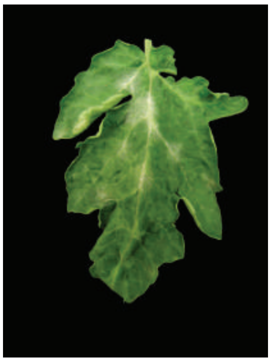
35S::CsaMLO8 WT - E

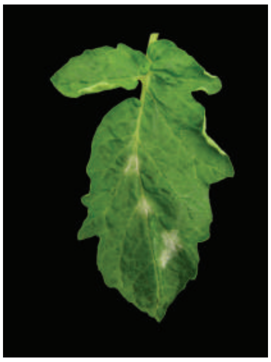
35S::CsaMLO8 WT - F

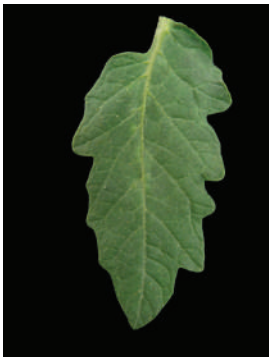
35S::CsaMLO8 WT - G

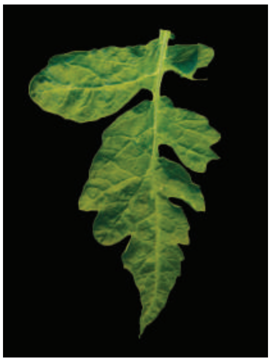
35S::CsaMLO8 WT - H

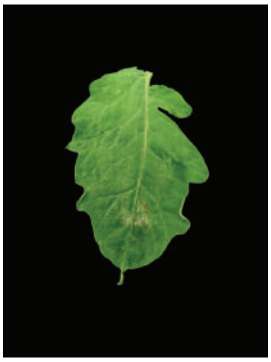
35S::CsaMLO8 WT - I

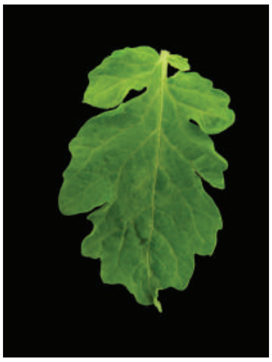
35S::CsaMLO8 WT - J

## *ol-2* 35S::*CsaMLO8* Δ174 transformants:

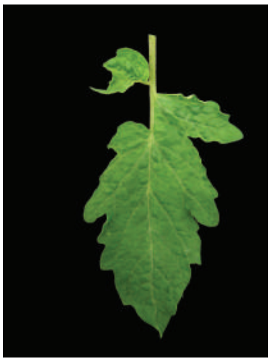
35S::CsaMLO8 Δ174 - A

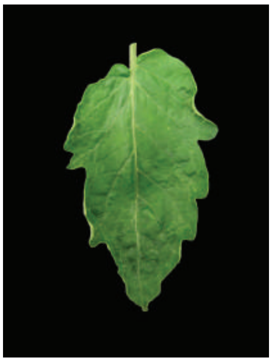
35S::CsaMLO8 Δ174 - B

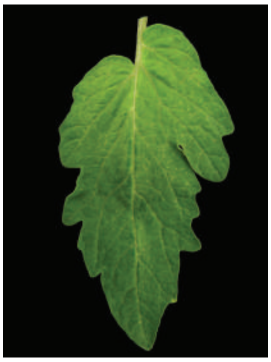
35S::CsaMLO8 Δ174 - C

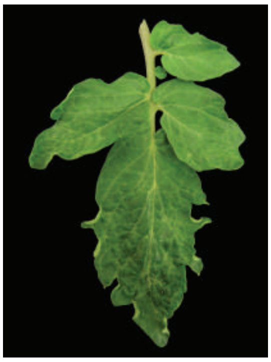
35S::CsaMLO8 Δ174 - D

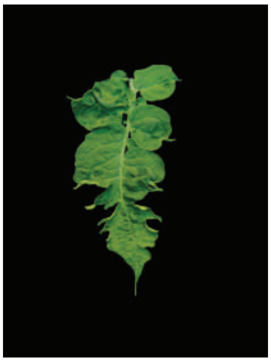
35S::CsaMLO8 Δ174 - E

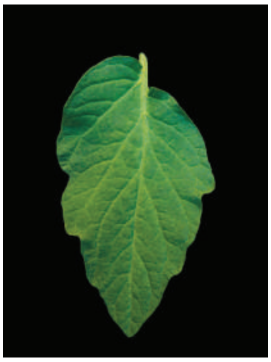
35S::CsaMLO8 Δ174 - F

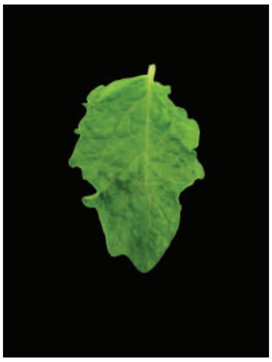
35S::CsaMLO8 Δ174 - G

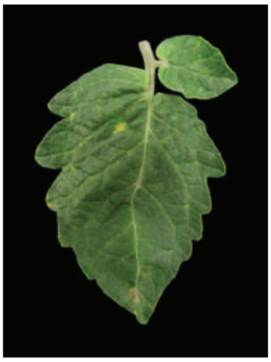
35S::CsaMLO8 Δ174 - H

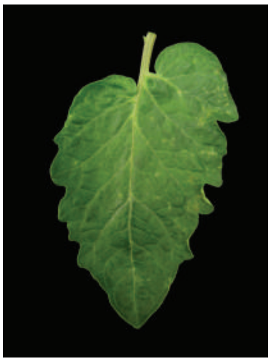
35S::CsaMLO8 Δ174 - I

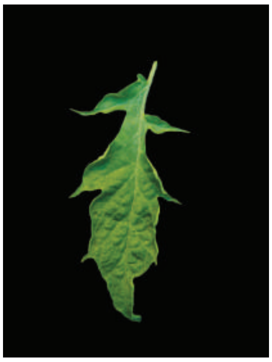
35S::CsaMLO8 Δ174 - J
