# Supplementary material for: A transposable element insertion in the susceptibility gene CsaMLO8 results in hypocotyl resistance to powdery mildew in cucumber
Source: BMC Plant Biol. 2015 Oct 9;15:243. doi: 10.1186/s12870-015-0635-x (PMC4600303; doi:10.1186/s12870-015-0635-x)
Supplement: Additional file 2: — Multiple sequence alignment of MLO proteins encoded by clade V MLO S -genes from different species. (PDF 287 kb) [file 12870_2015_635_MOESM2_ESM.pdf]

		20		40		
AtMLO2	MA-DQVKERT	LEETSTWAVA	VVCFVLLFIS	IVLEHSIHKI	GTWFKKKHKQ	49
AtMLO6	MA-DQVKEKT	LEETSTWAVA	VVCFVLLLIS	IVIEKLIHKI	GSGFKKKNKK	49
AtMLO12	MA---IKERS	LEETPTWAVA	VVCFVLLFIS	IMIEYFLHFI	GHWFKKKHKK	47
MtMLO1	MAEDKVYERT	LEETPTWAVA	VVCFVLLAIS	IVIEHIIHAI	GKWFKKKNKN	50
PsMLO1	MAEEGVKERT	LEETPTWAVA	VVCLVLLAVS	ILIEHIIHVI	GKWLKKRNKN	50
LjMLO1	M--DKVAQKK	LEETPTWAVA	VVCFVMLAIS	IIIEHGIEAI	EKWLEKRHKK	48
SlMLO1	M---------	-EATPTWAIA	VVCFILLAIS	IFIEQIIHHI	GEWLLEKRKK	40
CaMLO2	MA----KERS	MEATPTWAVA	VVCFILLAIS	IFIEQIMHHL	GEWLLKKHKK	46
NtMLO1	M---------	-EATPTWAVA	AVCFILLAIS	IFIEQIIHHL	GEWLLKKHKK	40
CsaMLO8	MAECGTEQRT	LEDTSTWAVA	VVCFFLVVIS	IFIEHVIHLT	GKWLEKRHKP	50
Consensus	MA-D-VKERT	LEETPTWAVA	VVCFVLLAIS	IFIEHIIHHI	GKWLKKKHKK	

	60		80		100	
AtMLO2	ALFEALEKVK	AELMLLGFIS	LLLTIGQTPI	SNICISQKVA	STMHPCSA-A	98
AtMLO6	ALYEALEKVK	AELMLMGFIS	LLLTIGQGYI	SNICIPKNIA	ASMHPCSA-S	98
AtMLO12	ALSEALEKVK	AELMLLGFIS	LLLVVLQTPV	SEICIPRNIA	ATWHPCSN-H	96
MtMLO1	ALYEALEKVK	GELMLMGFIS	LLLTVFQDYI	SKICISEKVG	STWHPCST-P	99
PsMLO1	ALYEALEKIK	GELMLLGFIS	LLLTVFQDNI	SKICVSQKIG	STWHPCST-S	99
LjMLO1	ALHEAVEKIK	GELMLMGFIS	FLLTVFKDPI	SNICISKQVA	STWHPCHP-E	97
SlMLO1	SLYEALEKIK	AELMLLGFLS	LLLTVLQDPV	SNLCVPKSVG	YSWHPCMAKE	90
CaMLO2	PLYEALEKIK	AELMLLGFIS	LLLTVIQDPV	SNLCVPKSVG	YSWHPCKADE	96
NtMLO1	PLYEALEKIK	AELMLLGFIS	LLLTVVQSPV	SNLCVPKSVG	YSWHPCKSDE	90
CsaMLO8	ALVEALEKVK	AELMLLGFIS	LLLTIGQDAV	TQICVSKELA	ATWLPCAARA	100
Consensus	ALYEALEKXK	AELMLLGFIS	LLLTVXQDPX	SNICXXKXVX	STWHPCSA-E	

		120		140		
AtMLO2	EEAKKYGKKD	AGKKDDGDGD	KPGRRLLLEL	AES---YIHR	RSLATKG---	142
AtMLO6	EEARKYGKKD	VPKEDE---E	ENLRRKLLQL	VDS---LIPR	RSLATKG---	139
AtMLO12	QEIAKYGKDY	I---DDG---	----RKILED	FDSNDFYSPR	RNLATKG---	133
MtMLO1	KT-----KT-	ASNDENSESE	-NHDRKLLEY	FDPN----PR	RILATKG---	135
PsMLO1	NT-----KAK	AKSDESLDYK	TNNDRKLLEY	FDPI----PR	RILATKG---	137
LjMLO1	EK-----K--	----------	----------	----------	-----KGPEG	105
SlMLO1	DAKSEYD---	----------	----------	----------	----------	97
CaMLO2	DVKSEYD---	----------	----------	----------	----------	103
NtMLO1	AAKNKYD---	----------	----------	----------	----------	97
CsaMLO8	KTGVKVAK--	----------	-NSRLRLLEF	LDPD--YGSR	RILASKGD--	133
Consensus	EXK-KYXK--	----------	----RKLLE-	-D------PR	R-LATKG---	

	160		180		200	
AtMLO2	-YDKCAEKGK	--VAFVSAYG	IHQLHIFIFV	LAVVHVVYCI	VTYAFGKIKM	189
AtMLO6	-YDKCAEKGK	--VAFVSAYG	MHQLHIFIFV	LAVCHVIYCI	VTYALGKTKM	186
AtMLO12	-YDKCAEKGK	--VALVSAYG	IHQLHIFIFV	LAVFHVLYCI	ITYALGKTKM	180
MtMLO1	-YDQCADKGK	--VALVSAYG	IHELHIFIFV	LAIFHILQCI	ITLALGRFKM	182
PsMLO1	-YDKCFDKGQ	--VALVSAYG	IHQLHIFIFV	LALFHILQCI	ITLTLGRIKM	184
LjMLO1	YYDKCAKDGK	DKVAFMSQYG	IHQLHIFIFV	LAIFHILQCI	TTLALGRTRM	155
SlMLO1	--DPCLPKGK	--VQFASSYA	IHQLHIFIFV	LAVAHVLYCI	ATFALGRLKM	143
CaMLO2	--DPCLQKGK	--VQFASSYA	IHQLHIFIFV	LAIAHVLYCI	ATFALGRLKM	149
NtMLO1	--DPCLPKGK	--VQFASSYA	IHQLHIFIFV	LAVAHVLYSI	ATFALGRLKM	143
CsaMLO8	--DACAKRGQ	--LAFVSAYG	IHQLHIFIFV	LAVFHVLYCI	ITLAFGRTKM	179
Consensus	-YDKCAEKGK	--VAFVSAYG	IHQLHIFIFV	LAVFHVLYCI	ITLALGRTKM	

		220		240		
AtMLO2	RTWKSWEEET	KTIEYQYSND	PERFRFARDT	SFGRRHLNFW	SKTRVTLWIV	239
AtMLO6	RRWKKWEEET	KTIEYQYSHD	PERFRFARDT	SFGRRHLSFW	SKSTITLWIV	236
AtMLO12	KKWKSWERET	KTIEYQYAND	PERFRFARDT	SFGRRHLNIW	SKSTFTLWIT	230
MtMLO1	RRWKKWEDET	RTVEYQFYND	PERFRFARDT	TFGRRHLSMW	TKSPISLWIV	232
PsMLO1	RKWKTWEDET	RTVEYQFYND	PERFRFARDT	TFGRRHLSMW	AQSPILLWIV	234
LjMLO1	AMWKKWEEET	KTLEHQFDND	PERFRFARDT	TFGRRHLNSW	SQSPISLWIV	205
SlMLO1	RKWRAWEDET	KTMEYQFYND	PERFRFARET	SFGRRHLHFW	SKSPVLLSIV	193
CaMLO2	RKWRAWEDET	KTIEYQFYND	PERFRFARET	SFGRRHMHFW	SKSPVMLWIV	199
NtMLO1	RKWRAWEEET	KTIEYQFYND	PERFRFARET	SFGRRHLHYW	SKSPVLLWIV	193
CsaMLO8	SKWKAWEDET	KTIEYQYYND	PARFRFARDT	TFGRRHLSFW	SRTPISLWIV	229
Consensus	RKWKAWEDET	KTIEYQFYND	PERFRFARDT	SFGRRHLSFW	SKSPIXLWIV	

	260		280		300	
AtMLO2	CFFRQFFGSV	TKVDYLALRH	GFIMAHFAPG	NESRFDFRKY	IQRSLEKDFK	289
AtMLO6	CFFRQFFRSV	TKVDYLTLRH	GFIMAHLAPG	SDARFDFRKY	IQRSLEEDFK	286
AtMLO12	CFFRQFFRSV	TKVDYLTLRH	GFIMAHLPAG	SAARFDFQKY	IERSLEQDFT	280
MtMLO1	CFFRQFFGSI	SRVDYLALRH	GFIMAHLAPG	NDAEFDFQKY	ISRSLEKDFK	282
PsMLO1	SFFRQFFGSI	SRVDYMALRH	GFIMAHLPPG	HDAQFDFQKY	ISRSIEEDFK	284
LjMLO1	SFFRQFYGSV	DKVDYMVLRH	GFIIAHLAPG	SESKFDFQKY	ISRSVDEDFK	255
SlMLO1	CFFRQFFSSV	AKVDYLTLRH	GFMMAHLTPQ	NQNNFDFQLY	INRAVDKDFK	243
CaMLO2	CFFRQFFSSV	AKVDYLTLRH	GFMMAHLTPQ	NQENFDFQIY	INRAVDKDFK	249
NtMLO1	CFFRQFFSSV	AKVDYLTLRH	GFMMAHLTPQ	NQENFDFQIY	INRAVEKDFK	243
CsaMLO8	CFFRQFFGSV	TKVDYMTLRH	GFIVAHLAPG	SEVKFDFHKY	ISRSLEDDFK	279
Consensus	CFFRQFFGSV	TKVDYLTLRH	GFIMAHLAPG	NXAXFDFQKY	ISRSLEKDFK	

		320		340		
AtMLO2	TVVEISPVIW	FVAVLFLLTN	SYGLRSYLWL	PFIPLVVILI	VGTKLEVIIT	339
AtMLO6	TIVEINPVIW	FIAVLFLLTN	TNGLNSYLWL	PFIPFIVILI	VGTKLQVIIT	336
AtMLO12	VVVGISPLIW	CIAVLFILTN	THGWDSYLWL	PFLPLIVILI	VGAKLQMIIS	330
MtMLO1	VVVGISPTIW	FFAVLFLLTN	THGWYSSYWL	PFLPLIIILL	VGAKLQMIIT	332
PsMLO1	VVVGISPTIW	LFTVLFLLTN	THGWYSYYWL	PFLPLIVILL	VGAKLQMIIT	334
LjMLO1	VVVGISPTVW	FFAVLILLTN	THGWHSYLWL	PFIPLVIILL	VGTKLQMIIT	305
SlMLO1	VVVGISPALW	LFTVLYFLTT	TDRLYSYLWV	PFIPLVIILL	VGTKLQMIIT	293
CaMLO2	VVVGISPALW	LFTVLYFLST	TDGVYSYLWV	PFVPLIIILL	VGTKLQMIIT	299
NtMLO1	FVVEISPALW	LFTVLYFLTT	TNGLYSYLWV	PFIPLVIILL	VGTKLEMIIA	293
CsaMLO8	VVVGISPAMW	LFAVLFILTN	TNGWYSYLWL	PFISLIIILL	VGTKLHVIIT	329
Consensus	VVVGISPAIW	LFAVLFLLTN	THGWYSYLWL	PFIPLIIILL	VGTKLQMIIT	

	360		380		400	
AtMLO2	KLGLRIQEKG	DVVRGAPVVQ	PGDDLFWFGK	PRFILFLIHL	VLFTNAFQLA	389
AtMLO6	KLGLRIQEKG	DVVKGTPLVQ	PGDHFFWFGR	PRFILFLIHL	VLFTNAFQLA	386
AtMLO12	KLGLRIQEKG	DVVKGAPVVE	PGDDLFWFGR	PRFILFLIHL	VLFTNAFQLA	380
MtMLO1	KMGLRIQDRG	EVIKGAPVVE	PGDHLFWFNS	PNLLLFIIHL	VLFQNAFQLA	382
PsMLO1	KMGLRIQDRG	EVIKGAPVVE	PGDHLFWFNR	PHLLLFTIHL	VLFQNAFQLA	384
LjMLO1	NMGLKIQERG	DVIKGAPLVE	PGDDLFWFNR	PRLILSLVHL	VLFQNAFQLA	355
SlMLO1	EMGVRISERG	DIVKGVPVVE	TGDHLFWFNR	PALVLFLINF	VLFQNAFQVA	343
CaMLO2	EMGVRISERG	DIVKGVPVVE	IGDHLFWFNR	PGLVLFFINF	VLFQNAFQVA	349
NtMLO1	EMGVRISKRG	DIVRGVPVVE	TGDHLFWFNR	PGFVLFLINF	VLFQNAFQVA	343
CsaMLO8	HMGLTIQERG	HVVKGVPVVQ	PRDDLFWFGR	PQLILFLIHF	VLFMNAFQLA	379
Consensus	KMGLRIQERG	DVVKGAPVVE	PGDHLFWFNR	PRLILFLIHL	VLFQNAFQLA	

		420		440		
AtMLO2	FFAWSTYE--	FNLNNCFHES	TADVVIRLVV	GAVVQILCSY	VTLPLYALVT	437
AtMLO6	FFVWSTYE--	FGLKNCFHES	RVDVIIRISI	GLLVQILCSY	VTLPLYALVT	434
AtMLO12	FFVWSTYE--	FTLKNCFHHK	TEDIAIRITM	GVLIQVLCSY	ITLPLYALVT	428
MtMLO1	FFSWSTYE--	FSINSCFHRT	TADNVIRVSV	GILIQFLCSY	VTLPLYALVT	430
PsMLO1	FFAWSTYE--	FSITSCFHKT	TADSVIRITV	GVVIQTLCSY	VTLPLYALVT	432
LjMLO1	FFAWSACDND	FKINSCFHRS	TADVVIRLTL	GVVTQVLCSY	VTLPLYALVT	405
SlMLO1	FFFWSWWK--	FGFPSCFHKN	AADLAIRLTM	GVIIQVHCSY	VTLPLYALVT	391
CaMLO2	FFVWSWWK--	FGFPSCFHRN	AADLAIRLTM	GVIIQVHCSY	VTLPLYALVT	397
NtMLO1	FFVWSWWK--	FSYPSCFHQN	AADIAIRLTM	GVIIQVHCSY	VTLPLYALVT	391
CsaMLO8	FFAWTTY--A	FKWMGCFHQR	VEDIVIRLSM	GVIIQVLCSY	VTLPLYALVT	427
Consensus	FFXWSTYE--	FXXXSCFHRX	TADXVIRLTM	GVIIQVLCSY	VTLPLYALVT	

	460		480		500	
AtMLO2	QMGSKMKPTV	FNDRVATALK	KWHHTAKNET	KHGRHSGS--	NTPFSSRPTT	485
AtMLO6	QMGSKMKPTV	FNERVATALK	SWHHTAKKNI	KHGRTSES--	TTPFSSRPTT	482
AtMLO12	QMGTSMRPTI	FNDRVANALK	KWHHTAKKQT	KHG-HSGS--	NTPHSSRPTT	475
MtMLO1	QMGSTMKPTI	FNERLATALK	KWHHTAKKQV	KHNKHSNN--	TTPYSSRQST	478
PsMLO1	QMGSTMKPTI	FNERVATALK	NWHHTAKKQV	KQSNHSNN--	TTPYSSRPST	480
LjMLO1	QMGSTMRPTI	FHDRVATALK	SWHHTAKKHV	KHNRDSNSHS	NTPFSSRPAT	455
SlMLO1	QMGSSMKPII	FGDNVATALR	SWHHTAKKRV	KHG-LSGH--	TTPANSRPTT	438
CaMLO2	QMGSSMKPII	FGDNVATALR	SWHNTAKKRV	RHGRVSEN--	TTPISSRPAT	445
NtMLO1	QMGTSMKPII	FGDNVATALR	SWHNTAKKRV	KHGRLSEN--	TTPVSSRPAT	439
CsaMLO8	QMGSNMRPTI	FNDRVATALK	NWHHSAKKNM	KQ--HRNPDS	TSPFSSRPAT	475
Consensus	QMGSSMKPTI	FNDRVATALK	SWHHTAKKXV	KHGRHSNX--	TTPFSSRPXT	

		520		540		
AtMLO2	PTHGSSPIHL	LHNF---NNR	SV-E--NYPS	SPSPR----Y	SGHGHHEHQF	525
AtMLO6	PTHGSSPIHL	LRNAPHKRSR	SVDE--SFAN	SFSPR----N	S-----DFDS	521
AtMLO12	PTHGMSPVHL	LHNY---NNR	SLDQQTSFTA	SPSPPRFSDY	SGQGHGHQHF	522
MtMLO1	PTHGMSPVHL	LH--------	---RQ-TFGN	SDSLQTSPRT	S--NYENEQ-	513
PsMLO1	PTHAMSPVHL	LH--------	---RH-TAGN	SDSLQTSPEK	S--DYKNEQ-	515
LjMLO1	PTHGMSPVHL	LH--------	---KHHNYHN	SDSPLASPRE	SPSNYETEQ-	493
SlMLO1	PLRGTSPVHL	LRGYPQ----	-------Y-N	EDSVQASPRT	SNV---ENEG	473
CaMLO2	PLRGTSPVHL	LRGYPK----	-------Y-N	EDNVQAYPRT	SNV---ENEG	480
NtMLO1	PLHGTSPVHL	LRSYPQ----	-------YSN	EES-----RT	SNA---ENEG	470
CsaMLO8	PTHGMSPIHL	LHKHQH----	---------G	STSPRLSDA-	------EPDR	505
Consensus	PTHGMSPVHL	LHNY------	-------Y-N	SDSPQASPRT	S-----EXQG	

	560		580		600	
AtMLO2	WDPESQHQE-	------AETS	T--HHS-LAH	ESSEP--VLA	SVELPP----	559
AtMLO6	WDPESQHET-	------AETS	NSNHRSRFGE	EESEKKFVSS	SVELPPGPGQ	564
AtMLO12	FDPESQNHSY	QREITDSEFS	NSHH------	----PQVDMA	S--------P	554
MtMLO1	WDVEGGGSTS	PRN-----NQ	TVASEIEIPI	VES-----FS	TTELP-----	548
PsMLO1	WDIEGEGPTS	LRN-----DQ	TGQHEIQIAG	VES-----FS	STELP-----	550
LjMLO1	WYLE---PNS	PSNHTRGHDQ	TLQMQVLGSS	ATE-----FS	----P-----	526
SlMLO1	WANEN-----	----------	----------	----------	----------	478
CaMLO2	WANETS----	----------	----------	----------	----------	486
NtMLO1	WANEIP----	----------	----------	----------	----------	476
CsaMLO8	WE--------	----------	----------	----------	--ELPPSSHH	515
Consensus	WDXESQ----	----------	T--H------	---------S	--ELP-----	

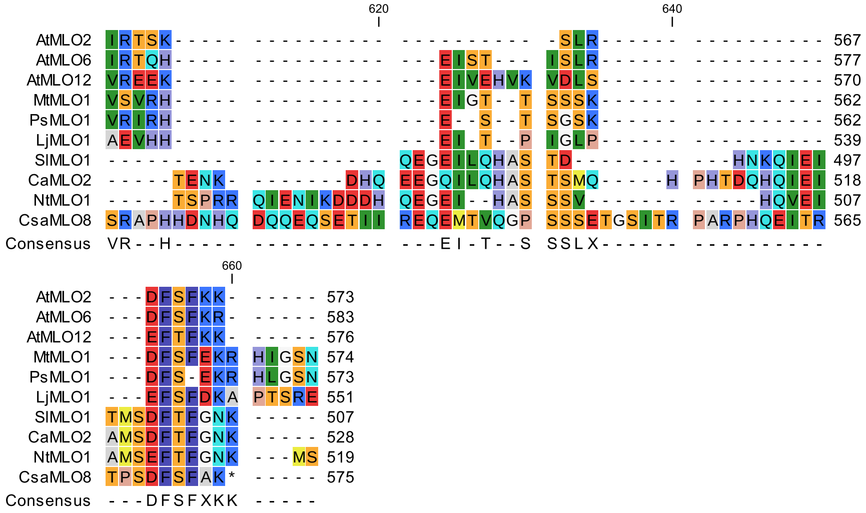

620
640
AtMLO2 IRTSK----- ---------- ---------- -SLR------ ---------- 567
AtMLO6 IRTQH----- ---------- ---EIST--- ISLR------ ---------- 577
AtMLO12 VREEK----- ---------- ---EIVEHVK VDLS------ ---------- 570
MtMLO1 VSVRH----- ---------- ---EIGT--T SSSK------ ---------- 562
PsMLO1 VRIRH----- ---------- ---E--S--T SGSK------ ---------- 562
LjMLO1 AEVHH----- ---------- ---EI-T--P IGLP------ ---------- 539
SlMLO1 ---------- ---------- QEGEILQHAS TD-------- ---HNKQIEI 497
CaMLO2 -----TENK- -------DHQ EEGQILQHAS TSMQ-----H PHTDQHQIEI 518
NtMLO1 -----TSPRR QIENIKDDDH QEGEI--HAS SSV------- -----HQVEI 507
CsaMLO8 SRAPHHDNHQ DQQEQSETII REQEMTVQGP SSSETGSITR PARPHQEITR 565
Consensus VR--H----- ---------- ---EI-T--S SSLX------ ----------
660
AtMLO2 ---DFSFKK- ----- 573
AtMLO6 ---DFSFKR- ----- 583
AtMLO12 ---EFTFKK- ----- 576
MtMLO1 ---DFSFEKR HIGSN 574
PsMLO1 ---DFS-EKR HLGSN 573
LjMLO1 ---EFSFDKA PTSRE 551
SlMLO1 TMSDFTFGNK ----- 507
CaMLO2 AMSDFTFGNK ----- 528
NtMLO1 AMSEFTFGNK ---MS 519
CsaMLO8 TPSDFSFAK* ----- 575
Consensus ---DFSFXKK -----
